# Tumor-targeting bioluminescent bacteria for *in vivo* imaging

**DOI:** 10.1016/j.engmic.2025.100224

**Published:** 2025-07-11

**Authors:** Chenghao Ma, Jingxi Liu, Hongjing Liu, Xiaohan Zhao, Geng Li, Youming Zhang, Tianyu Jiang

**Affiliations:** a State Key Laboratory of Microbial Technology, Institute of Microbial Technology, Helmholtz International Lab for Anti-Infectives, Shandong University–Helmholtz Institute of Biotechnology, Shandong University, Qingdao 266237, China; b Faculty of Pharmacy and Pharmaceutical Sciences, Monash Institute of Pharmaceutical Sciences, Monash University, Melbourne, Victoria 3052, Australia; c School of Life Sciences, Shandong University, Qingdao 266237, China; d Shenzhen Research Institute of Shandong University, Shenzhen, Guangdong 518000, China

**Keywords:** *Escherichia coli* Nissle 1917, *In vivo* bioluminescence imaging, Luciferase, Coelenterazine

## Abstract

As the understanding of bacteria-mediated cancer therapies deepens, bacteria such as *Escherichia coli* Nissle 1917 (EcN) have become a promising platform for cancer therapy. However, their potential role in real-time monitoring and visualization tools still needs to be explored and enhanced. In this study, we aimed to screen and optimize EcN visualization systems for non-invasive *in vivo* bioluminescence imaging in live mice. To this end, we developed three series of recombinant EcN strains expressing *Gaussia* luciferase (Gluc), *Renilla* luciferase (Rluc), and NanoLuc (Nluc), along with their respective mutants. These strains exhibited bioluminescence when different coelenterazine (CTZ) substrates were present. As a result, multiple bioluminescent EcN strain–substrate pairs were identified with stronger, longer, or red-shifted bioluminescence, offering multiple effective optical tumor-targeting systems for *in vivo* studies investigating bacteria-mediated cancer therapy and intestinal diseases.

## Graphical abstract

**Figure:**
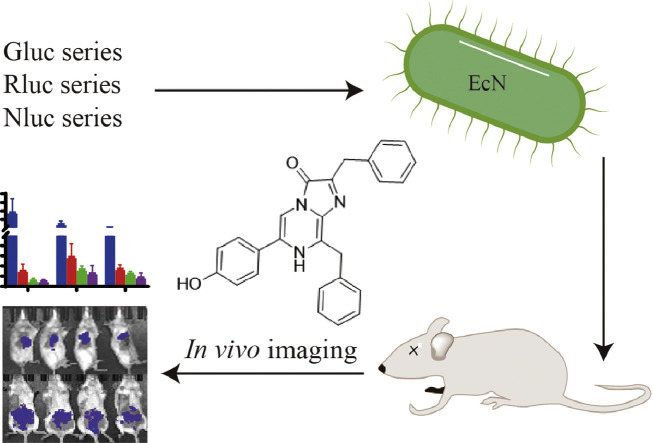

## 1 Introduction

Several therapeutic approaches, including bacteria-mediated treatment, have recently been developed and optimized to achieve effective cancer therapy with low side effects and recurrence rates [[1], [2], [3]]. Bacteria-mediated cancer therapy can be integrated with synthetic biology, immunology, and other treatment options for targeted cancer therapy [[4], [5], [6]]. A probiotic bacterium, *E. coli* Nissle 1917 (EcN), has shown safe and non-toxic properties, with a high affinity and the ability to proliferate extensively in solid tumors, making it a promising bacterial chassis for tumor therapy. To better understand the biological processes involved in bacterial tumor targeting, it is necessary to develop real-time and *in vivo* bacterial imaging methods to monitor and determine bacterial colonization. Bioluminescence imaging (BLI) is a powerful tool that utilizes engineered bioluminescent bacteria to visualize and track biological processes both *in vivo* and *in vitro*. Building upon this foundational insight, BLI has emerged as a non-invasive alternative to conventional biopsy methods, significantly reducing patient discomfort and expediting diagnosis times. More importantly, the capacity to monitor bacterial activity and efficacy within tumors introduces a novel dimension to cancer treatment assessments, paving the way for a refined understanding of bacteria-mediated therapies [7]. Hence, engineered bioluminescent EcN strains are ideal targeting probes for cancer imaging.

However, the availability of visualization tools has limited the research on EcN-based cancer therapeutics. In tumor-targeting bacterial research, the lux receptor and firefly luciferase (Fluc) bioluminescence system are commonly used to monitor and track bacterial behavior in cancer therapy studies. For instance, a programmable EcN consisting of a genomically integrated *luxCDABE* operon and a plasmid with high *lacZ* expression has been demonstrated to monitor and track *in vivo* bacterial migration to tumors [8]. Similarly, the novel EcN strain genomically integrated with the Fluc-luciferin regenerating enzyme (LRE) system shows the ability to generate continuous red-shifted light for improved *in vivo* analytic imaging tools for bacterial cancer therapy [9].

However, the *luxCDABE* bioluminescence reporter gene has weak luminescence intensity and a short wavelength, leading to poor sensitivity and penetration factor in live tissue imaging [10,11]. Therefore, diverse bioluminescence tools should be developed for enhancing the imaging of the tumor-targeting process of bacteria.

**Scheme 1:**
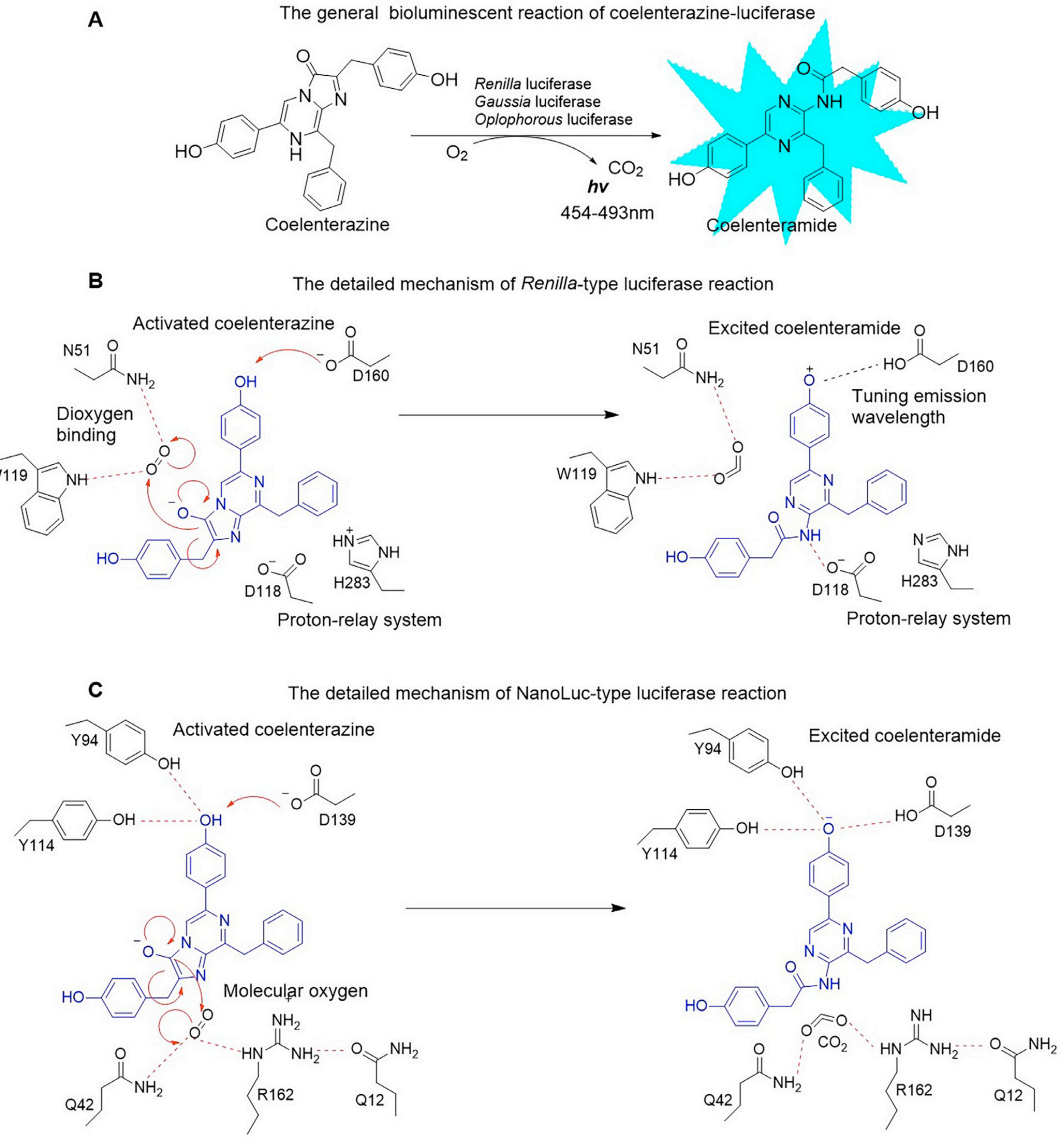
**The mechanism of CTZ-type bioluminescence.** (A) The general reaction mechanism of CTZ-luciferase bioluminescence. (B) The detailed mechanism of *Renilla*-type luciferase reaction based on the crystal structure of *Renilla* luciferase [25]. (C) The detailed mechanism of Nanoluc-type luciferase reaction based on the crystal structure of Nanoluc [26].

As research on bioluminescence systems advances, the luciferase–luciferin system has been widely used as a modelling tool in life sciences. Coelenterazine (CTZ)-dependent bioluminescence encompasses two types of proteins: luciferases and photoproteins. Their bioluminescence mechanisms are different [12]. Luciferase can oxidize CTZ without cofactors, generating coelenteramide (the decarboxylated derivative of CTZ), CO₂, and a quantum of blue light (Scheme 1A). In contrast, CTZ-dependent photoproteins rely on Ca²⁺. A Ca²⁺-regulated photoprotein is formed when the polypeptide apophotoprotein oxidizes CTZ. Then, when the Ca²⁺-regulated photoprotein binds to Ca²⁺, bioluminescence occurs along with the generation of reaction products. In this study, we focused on CTZ-dependent luciferases and CTZ analogs. Various luciferase–luciferin combinations without the need for cofactors are available, thereby providing diversified imaging tools (Table 1). This unique property not only enriches the application scenarios of bioluminescence in the field of imaging but also offers new possibilities for in-depth research in related areas. CTZ-dependent luciferases include luciferases from marine organisms such as *Renilla, Gaussia princeps*, and *Oplophorus gracilirostris* (Oluc). Several mutant versions of Rluc have improved properties, including Rluc8 with an enhanced intensity [13,14], Rluc8.6 with a red-shift spectrum [15], and m6-Rluc with increased thermal stability [16] in the presence of CTZ. The Gluc and CTZ systems exhibit relatively optimal bioluminescence intensity among wild-type bioluminescence systems. Additionally, enhanced slGluc [17,18], sbGluc [19], and Gluc M23 [20] have been developed to achieve better bioluminescence intensity in the presence of CTZ. Mutant Nluc, derived from Oluc, produces bioluminescence 150 times stronger than that of Fluc or Rluc, with high thermal stability. Mutants of Nluc, such as Qluc [21], teNluc [22], and Antares [23], show favorable bioluminescence properties for adapting deep-tissue imaging in the presence of CTZ analogs such as furimazine (FRZ, a CTZ analog developed by Promega) or others. Luciferase from *Gaussia princeps* can react with CTZ to produce strong blue light, higher than that of Nluc and 200 times greater than that of Fluc or Rluc [24].

**Table 1:** Bioluminescence properties of various luciferase–luciferin pairs reported.

	Special activity	Peak of Wavelength	% > 600 nm	References
*Rluc series*				
Rluc-CTZ	1^a^	481	3	[15]
Rluc8-CTZ	4.3^a^	486	4	[15]
Rluc8.6–535-CTZ	6^a^	535	17	[15]
m6-Rluc-CTZ	1.23^a^	N/A	N/A	[16]
*Gluc series*				
Gluc-CTZ	1^a^^,^^b^	470	N/A	[[17], [18], [19], [20]]
slGluc-CTZ	∼3^b^	481	N/A	[17,18,20]
sbGluc-CTZ	∼6^b^	∼470	N/A	[17,19,20]
GlucM23-CTZ	∼6.5^b^	N/A	N/A	[20]
*Nluc series*				
Nluc-CTZ	∼1 a^,^^b^	478	N/A	[19,30]
Nluc-FRZ	∼4 b	455	2	[17,21]
Qluc-FRZ	∼ 3.5^a^	∼ 455	N/A	[21]
Antares-FRZ	∼2.72^a^	456 and 583	17	[22]

a Intensity values normalized to purified wild type luciferase-CTZ under comparable experimental conditions. All luciferases are purified proteins.

b Intensity values normalized to mammalian cell line expressing wild type luciferase in the presence of CTZ under comparable experimental conditions. All luciferases are expressed in mammalian cell line.

The crystal structures of Rluc [25], Nluc [26], and Gluc [26] have recently been resolved, contributing to our understanding of the mechanism of the reaction (Scheme 1B and 1C). Regarding the binding of luciferases, such as Rluc and Nluc, the -OH group of the C6-(p-hydroxyphenyl) substituent of CTZ is crucial for the formation of the activated dianion. The C2 carbon position of CTZ is also crucial for its involvement in attacking the side chains of luciferase. Although the structure of Gluc does not show an obvious substrate-binding pocket, given that Gluc's capacity to generate light through the oxidation of imidazopyrazinone derivatives is restricted to CTZ, the 2-hydroxybenzyl substituent or the -OH group of the C6-(p-hydroxyphenyl) substituent of CTZ must engage in essential interactions with the protein [27]. The hydrophobicity of the CTZ molecule allows it to penetrate the cell membrane. Thus, CTZ is a popular luciferin for *in vivo* bioluminescence imaging. Based on previous research findings, a vast array of analogs centered around the CTZ skeleton have been developed. These compounds are predominantly modified at the C2, C6, and C8 positions of the CTZ mother nucleus. Notably, numerous molecules with outstanding bioluminescence properties have emerged within this group. For example, FRZ, which has a furan-2-ylmethyl group at the C2 position of the CTZ skeleton, serves as an excellent substrate for Nluc [28]. Some C6-substituted CTZ derivatives like coelenterazine B5 (CTZ B5), coelenterazine B12 (CTZ B12), and coelenterazine h (CTZ h) have shown excellent bioluminescence activity and sustainability with Rluc [29].

Combining CTZ compounds with various types of luciferases can form bioluminescence visualization tools with high bioluminescence intensity, long duration, and shifted spectra (red or blue), with the aim of applying them for different research purposes. Increased bioluminescence intensity improves the sensitivity and accuracy of *in vivo* imaging, where red-shifted wavelengths can facilitate deep tissue imaging. This highlights that CTZ bioluminescence systems have a strong potential for applications in bacterial tumor-targeting research.

Therefore, this study aimed to address the lack of efficient bioluminescence imaging tools for bacteria-mediated cancer therapy studies. Given that various CTZ-dependent luciferase–luciferin pairs function as tools for imaging mammalian cells in mouse models, CTZ-type bioluminescent tools have the potential to image bacteria *in vivo*. EcN was utilized as the host to construct a CTZ-based bioluminescence visualization system to create diverse EcN tumor-targeted imaging models and develop highly sensitive tools with high bioluminescence intensity and wavelength red shift. This provides a variety of adaptable visualization systems for EcN to act as an anti-tumor drug in tumor-targeting expression and delivery systems. Moreover, this study explored and revealed the bacterial potential for tumor targeting and the delivery and expression of anti-tumor drugs. Bioluminescence imaging will facilitate the development of tumor-targeting bacteria, offering new strategies and methods for developing bacteria for tumor-targeting therapy.

## 2 Materials and methods

### 2.1 Strains, culture condition, and plasmids

Tables S1–S3 list the bacterial strains, plasmids, and primers used in this study. The *Escherichia coli* (*E. coli*) strains were cultured in Luria-Bertani (LB) medium at 37°C in a thermostatic oscillator at 950 rpm. The liquid LB medium consisted of 5 g/L yeast extract, 10 g/L pancreatin, and 1 % sodium chloride. Solid LB medium was prepared by adding 1.2 % agar to the liquid LB medium. Sodium chloride was provided by Genview, while the rest were supplied by Thermo Fisher.

### 2.2 Construction of plasmid and recombinant strains

Genewiz synthesized the partial fragments of the core genes, while the original backbone plasmid, pSB1C3-lac-RFP-CmR, was preserved in our laboratory. Some plasmids were constructed using the linear plus circular homologous recombination (LCHR) method. Initially, we amplified the luciferase and amp-ccdB forward-reverse selection elements, which were then fused using fusion PCR. The plasmid pSB1C3-cm and PCR products were electroporated into the genetically engineered strain *E.coli* GBred-gyrA462 to mediate homologous recombination. The correct plasmids were cleaved by a single restriction enzyme to recover using sodium acetate–ethanol precipitation. The recovered fragments were ligated using T4 ligase and electroporated into the genetically engineered strain *E.coli* GB2005, followed by T-streaking on LB medium plates containing chloramphenicol for reverse selection. The correct single colonies were identified using restriction enzyme digestion and sequenced.

The linear plus linear homologous recombination (LLHR) method was used to construct other plasmids. We amplified the luciferase and pSB1C3-cm parts. Following this, we used the MultiF Seamless Assembly Mix to mediate and verify homologous recombination. The plasmids constructed and primers used are shown in Table S1. Subsequently, recombinant EcN strains were generated using these plasmids (Fig. S1).

### 2.3 Bioluminescence properties of engineered strains

Referring to the determined optimal lysis OD_600_ and compound working concentration, the selected compounds CTZ, CTZ h, CTZ B12, CTZ B5, and FRZ were prepared as 50 µM and 100 µM solutions, as shown in Table 2.

**Table 2:** Compounds and corresponding buffers required for the study.

Structures of CTZ compounds		

Strains	Chemical compounds (50/100 µM)	Buffers
Rluc series	CTZ, CTZ h, CTZ B5, CTZ B12	Tris–HCl (50 mM, pH 7.42)
Gluc series	CTZ, CTZ h, CTZ B5, CTZ B12	Tris–HCl (25 mM, pH 7.8, including 600 mM NaCl, 1 mM EDTA, 0.05 % BSA)
Nluc series	FRZ	Tris–HCl (50 mM, pH 7.42)

We first cultured the bacteria to be tested overnight to determine the luminescence intensity of the lysis solution. The OD_600_ was then measured, and the cultures were homogenized to an OD_600_ of 0.8 using the corresponding buffer. Bacterial samples (40 µL) were added to a transparent 96-well plate with 50 µL of the corresponding buffer and 10 µL of another buffer (1 M K_2_HPO_4_, 20 mM EDTA, pH 7.8). Subsequently, 300 µL of lysis solution (the volume ratio of lysozyme to 2 × Cell Culture Lysis Reagent (CCLR) with 5 mg/mL BSA is 1:2) was added to each well, mixed thoroughly, and incubated at room temperature for 10 min. Then, 50 µL of lysed bacteria was transferred to a black 96-well plate with 50 µL of the corresponding compound, followed by the use of GloMax™ 96 Microplate Luminometer to immediately measure and confirm the initial luminescence intensity of each reaction mixture. We then meticulously quantified the luminescence intensity at discrete time intervals for each group using our validated method, which enabled a precise delineation of the bioluminescence duration.

To analyze the changes in the emission wavelength of the combinatorial screening, an instrument for spectral scanning was required for detection. The overnight-cultured bacteria were collected, dissolved in the corresponding buffer, and diluted to an OD_600_ of 0.8. After lysis, 100 µL of the culture was added to a 96-well plate with the compounds for testing. The mixture of lysed bacterial solution and compound was detected via the Synergy H1 fully functional microplate reader with a detection wavelength of 350–650 nm and a bioluminescence intensity detection step size of 5 nm. Finally, the spectrum was plotted using GraphPad Prism.

To test the luminescence ability of the EcN-Gluc series, we chose CTZ and DeepBlueC as substrates for condition optimization. The appropriate conditions were bacterial culture with an OD_600_ of 0.8 and substrates at a final concentration of 25 µM (Fig. S2). Using the same strategy, we tested the luminescence ability of the EcN-Rluc series combined with CTZ and DeepBlueC. The EcN-Rluc group had significantly higher luminescence intensity than the Gluc group. The maximum luminescence intensity of the EcN-Rluc 8.6 strain reached 5 × 10^7^ RLU at an OD_600_ of 0.8. The mutant's bioluminescence intensity was 10^3^ times higher than that of the EcN-Rluc series. The luminescence intensity trend of the EcN-Rluc group was positively correlated with the substrate concentration under the compound concentration gradient test (Fig. S3). Again, we tested the luminescence ability of the EcN—Nluc strain combined with the EcN-teNluc strain and FRZ. The luminescence intensity of both the EcN—Nluc and EcN-teNluc strains reached 2 × 10^7^ RLU at an OD_600_ of 0.8. The luminescence intensity trend of the EcN—Nluc group was also positively correlated with the substrate concentration under the compound concentration gradient test (Fig. S4).

### 2.4 In vivo imaging

All animal studies followed the guidelines of the Ethics Committee and the IACUC protocols of the School of Life Sciences, Shandong University. The experimental animals used in this study were 8-week-old female Balb/C mice obtained from Shandong Jinan Pengyue Animal Co., Ltd. Upon arrival, the mice were acclimatized in a controlled environment with a strict 12-hour light/dark cycle and provided sterile bedding, feed, and water for a week to stabilize their conditions. Later, we established the mouse allograft tumor model by subcutaneously injecting 10^5^ 4T1 murine breast cancer cells into the right forelimb. When the tumor grew to 300–600 mm^3^, we administered 100 µL of diluted EcN (OD_600_ = 0.2) containing the visualizable plasmid solution to the mice via tail vein injection. After 48 h, the mice were anesthetized using isoflurane, and the corresponding substrate solution was injected into the tumor. A small animal *in vivo* imaging system (IVIS Spectrum) was used in automatic exposure mode for *in vivo* imaging detection.

For intestinal tracing, after the mouse was stable, the mouse was starved for 16 h, then gavaged with 400 µL of EcN (OD_600_ = 0.8) containing the corresponding visualization plasmid and was intraperitoneally injected with a solution containing the corresponding substrate 2 h later. For immediate *in vivo* imaging, the IVIS Spectrum was used with automatic exposure.

## 3 Results and discussion

In our study, we heterogeneously expressed CTZ-dependent luciferases, including those from the Rluc series (Rluc, Rluc8, Rluc8.6, m6-Rluc), Gluc series (Gluc, slGluc, sbGluc, GlucM23), and Nluc series (Nluc, teNluc, Qluc, Antares), in EcN to generate recombinant bioluminescent EcN. Subsequently, we combined CTZ-type substrates, such as CTZ, CTZ h, CTZ B5, CTZ B12, and FRZ, to conduct bacterial imaging both *in vitro* and *in vivo*. Our aim was to provide a variety of adaptable visualization systems for EcN, offering bioluminescence imaging analysis to explore and reveal the potential of tumor-targeting bacteria in cancer therapy.

### 3.1 Bioluminescence properties of engineered EcN strains containing Gluc series luciferases

**Fig. 1:**
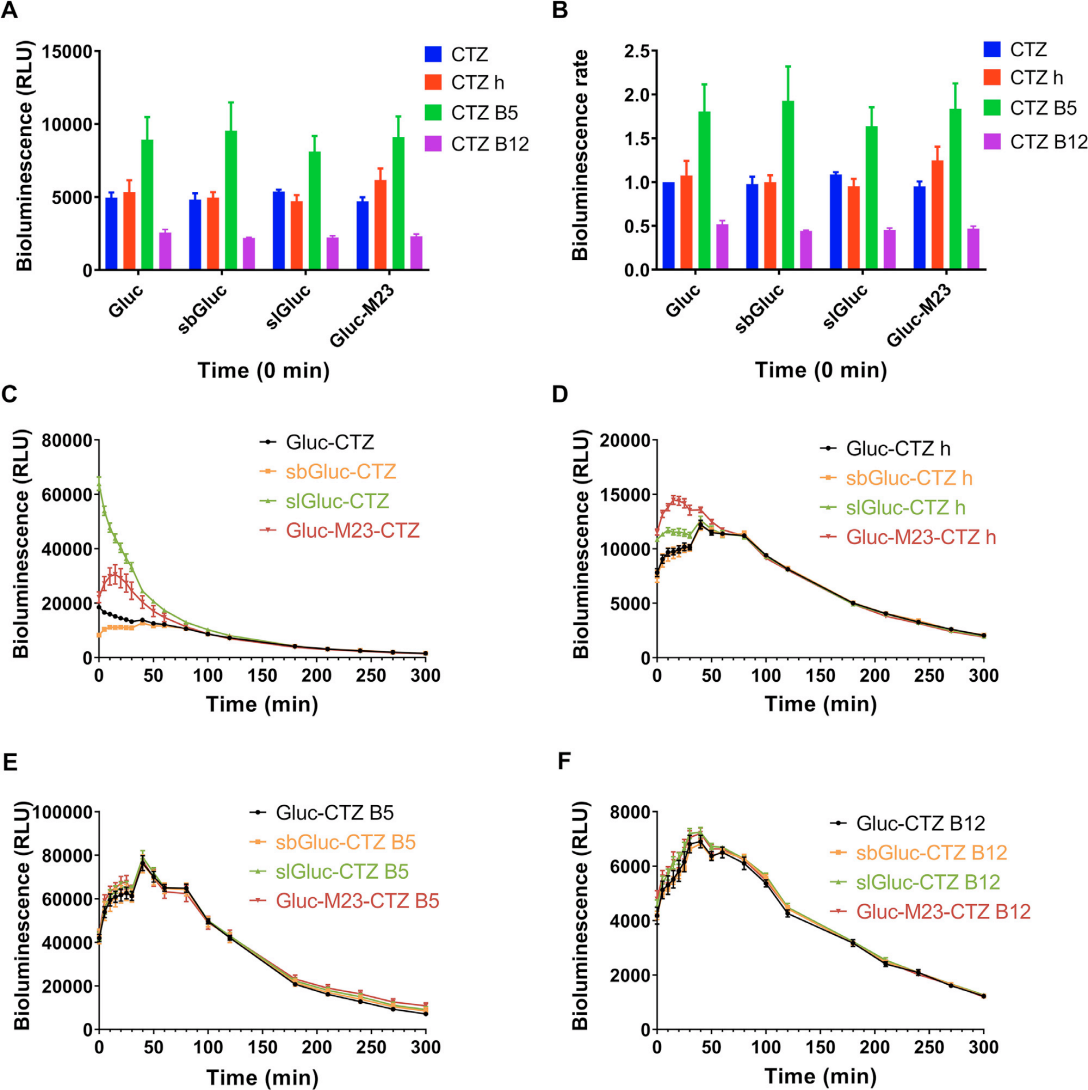
**Bioluminescence properties of engineered EcN strains containing Gluc Series luciferases.** Bioluminescence intensity of the Gluc series EcN strains with 25 µM CTZ analogs (A). Bioluminescence rate of the Gluc series EcN strains with 25 µM CTZ analogs (Gluc + CTZ set to 1) at 0 min (B). Bioluminescence time course of the engineered EcN strains containing Gluc series luciferases in the presence of CTZ (C), CTZ h (D), CTZ B5(E), CTZ B12 (F). The assay was performed in triplicate and is represented as the mean ± standard error of the mean (SEM).

We utilized the engineered EcN strain containing a Gluc luciferase series, including Gluc, sbGluc, slGluc, and Gluc M23, and CTZ and its analogs (CTZ h, CTZ B5, CTZ B12) to investigate the bioluminescence properties. The data were analyzed and plotted to obtain Fig. 1. In the CTZ group, the luminescence intensity ranked from highest to lowest at the beginning was EcN-slGluc, EcN-Gluc M23, EcN-Gluc, and EcN-sbGluc. As the reaction progressed, the luminescence intensities of Gluc and slGluc sharply decreased. By approximately 180 min, the luminescence intensity declined to a low level and remained stable (Fig. 1C). In the CTZ h group, the initial luminescence intensities of EcN-Gluc M23 and EcN-slGluc were slightly higher, followed by those of EcN-Gluc and EcN-sbGluc. The EcN-slGluc, EcN-Gluc, and EcN-sbGluc all reached their peak luminescence intensities post 15 min, while EcN-Gluc M23 peaked at 40 min. By 270 min, the luminescence intensities dropped to a low level and stabilized (Fig. 1D). In the CTZ B5 and CTZ B12 groups, the initial luminescence intensities of EcN-Gluc, EcN-slGluc, EcN-sbGluc, and EcN-Gluc M23 were comparable. At approximately 40 min, their luminescence intensities peaked and became similar. The luminescence intensities for CTZ B5 and CTZ B12 decreased to low levels at approximately 240 min and 270 min, respectively, and subsequently stabilized (Fig. 1E, F). However, the bioluminescence spectral data for the Gluc series strains interacting with CTZ analogs remained undetectable, potentially due to the bioluminescence signals being too weak to be captured by the high-sensitivity spectroscopy equipment utilized.

According to the experimental results, the EcN strains harboring Gluc series luciferases demonstrated relatively strong bioluminescence intensity. Under the action of CTZ h and CTZ B5, they had a long-lasting and stable luminescence process. These advantages could enhance the imaging effects of these combinations in animals, which was confirmed by subsequent animal experiments (Figs. 8, 12). In previous studies, Gluc-type luciferases seemed to be limited to CTZ for light generation. However, our results indicated that CTZ h and CTZ B5 were more efficient than CTZ. CTZ h lacks an -OH group at the 2-position of the phenol ring in its skeleton, while CTZ B5 has a furyl group at the C6 substituent of its skeleton and no -OH group at the 2-position of the phenol ring. Although the crystal structure of Gluc binding to the substrate was not clearly resolved, we speculated that the -OH group at the 2-position of the CTZ skeleton did not interact essentially with the protein, whereas the furyl group at the 6-position of the CTZ skeleton might easily interact with the protein.

### 3.2 Bioluminescence properties of engineered EcN strains containing Rluc series luciferases

**Fig. 2:**
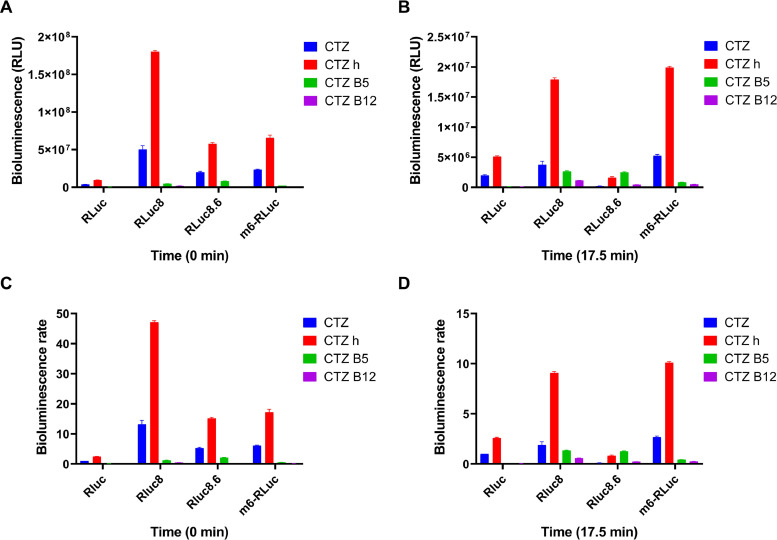
**Bioluminescence intensity of engineered EcN strains containing Rluc Series luciferases.** Bioluminescence intensity of the Rluc series EcN strains with 25 µM CTZ analogs at 0 min (A) and 17.5 min (B). Bioluminescence rate of the Rluc series EcN strains with 25 µM CTZ analogs (Rluc + CTZ set to 1) at 0 min (C) and 17.5 min (D). The assay was performed in triplicate and is represented as the mean ± standard error of the mean (SEM).

We utilized the engineered EcN strain containing Rluc luciferase series, including Rluc, Rluc 8, Rluc 8.6, and m6-Rluc and CTZ and its analogs (CTZ h, CTZ B5, CTZ B12, DeepBlueC) to evaluate the bioluminescence activity. We first tested the luminescence intensity after reacting different luciferases with different CTZ and its derivatives. Subsequently, we analyzed and compared the luminescence intensity of the same luciferase under different substrates and different luciferases under the same substrate (Fig. 2A, B). Overall, under these conditions, EcN-Rluc 8.6 performed better in terms of luminescence intensity in the CTZ B5 group, while EcN-Rluc 8 showed better luminescence than the other three luciferases under the actions of the other three substrates. First, the combination of CTZ h with luciferases produced the best luminescence effect, with intensities nearly several times, and even tens of times, more significant than those of other substrates. Second, CTZ demonstrated good luminescence but was still inferior to the previous one. Among these, the combination of EcN-Rluc 8 and CTZ h performed best (Fig. 2). At 17.5 min, the luminescence of EcN-m6-Rluc combined with CTZ and CTZ h showed a better luminescence effect than that of EcN-Rluc8, further indicating the excellent persistence of EcN-m6-Rluc (Fig. 2B, D).

**Fig. 3:**
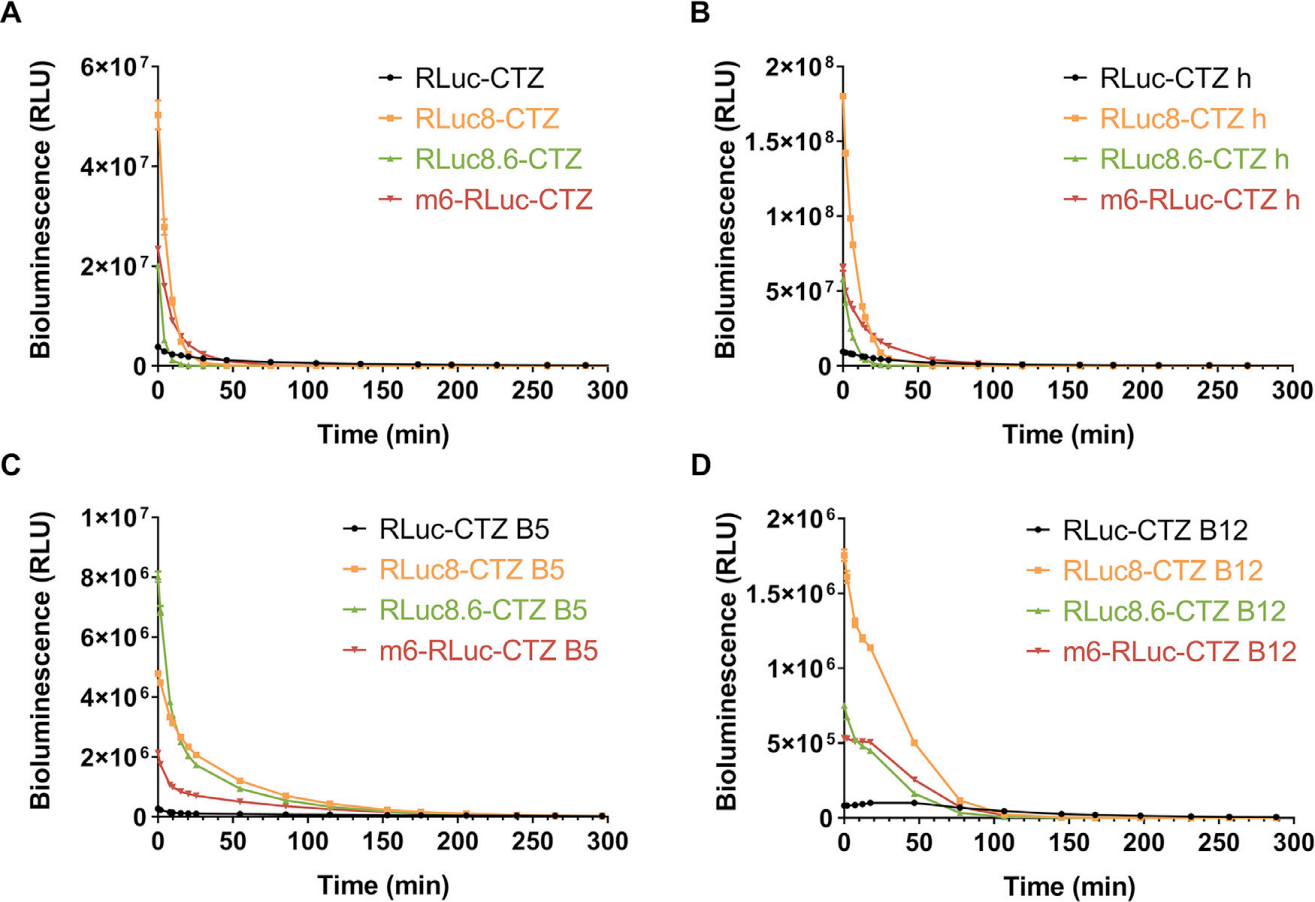
**The bioluminescence persistence of engineered EcN strains containing Rluc Series luciferases.** Bioluminescence time course of the engineered EcN strains containing Rluc series luciferases in the presence of CTZ (A), CTZ h (B), CTZ B5(C), CTZ B12 (D). The assay was performed in triplicate and is represented as the mean ± standard error of the mean (SEM).

In the CTZ and CTZ h groups, the luminescence intensity at the beginning ranked from highest to lowest was EcN-Rluc 8, EcN-m6-Rluc, EcN-Rluc 8.6, and EcN-Rluc. As the reaction progressed, the luminescence intensity sharply decreased, and after approximately 20 min, all the luminescence intensities had declined to a low level and remained stable (Fig. 3A, B). In the CTZ B5 group, the initial luminescence intensities were ordered from highest to lowest as follows: EcN-Rluc 8.6, EcN-Rluc 8, EcN-m6-Rluc, and EcN-Rluc. Later, their luminescence intensities gradually decreased and reached a low level at approximately 50 min, after which they remained stable (Fig. 3C). In the CTZ B12 group, the initial luminescence intensities were ranked as follows: EcN-Rluc 8, EcN-Rluc 8.6, EcN-m6-Rluc, and EcN-Rluc. Notably, the luminescence intensities of both EcN-Rluc and CTZ B12 initially increased before exhibiting a declining trend as the reaction progressed. The peak luminescence intensity occurred at approximately 18 min and then gradually decreased. By approximately 75 min, the luminescence intensities of all four EcN-expressing Rluc series luciferase strains had decreased to a low level and subsequently stabilized (Fig. 3D).

**Fig. 4:**
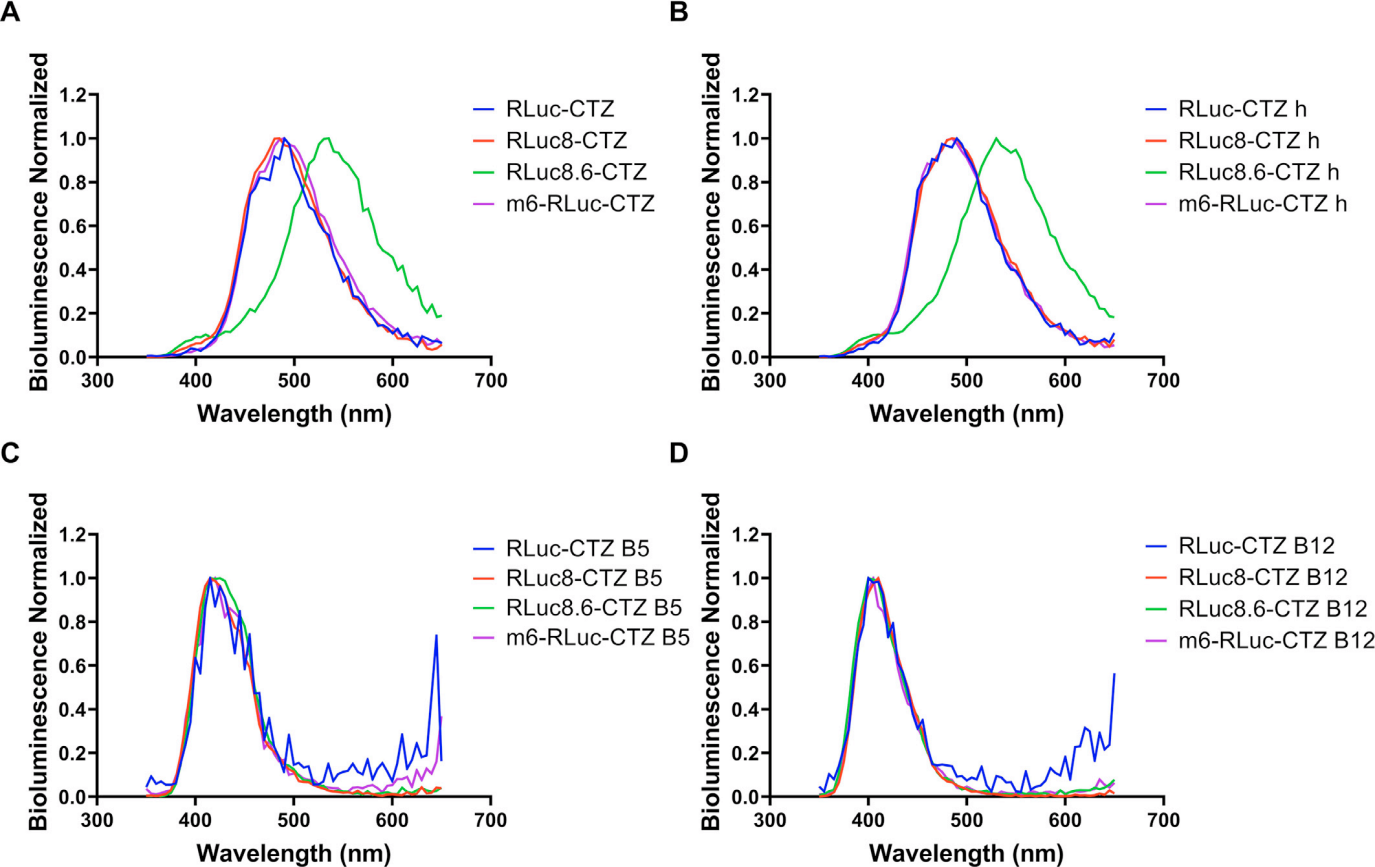
Bioluminescence emission spectra of the engineered EcN strains containing Rluc series luciferases under the action of CTZ (A), CTZ h (B), CTZ B5 (C), and CTZ B12 (D).

The maximum light intensity was normalized to 1 for plotting the spectra to measure the bioluminescence spectra of the EcN-Rluc strains combined with their corresponding substrates. We observed that the maximum emission wavelengths of the EcN-Rluc strains with the action of CTZ and CTZ h were approximately 490 nm, showing preferable luminescence performance (Fig. 4A, B). In contrast, the maximum emission wavelengths for CTZ B5 and CTZ B12 were lower, at approximately 415 nm (Fig. 4C, D). In particular, the maximum emission wavelengths of the EcN-Rluc 8.6-CTZ and EcN-Rluc 8.6-CTZ h combinations reached 535 nm and 530 nm, respectively, indicating a red shift of approximately 50 nm. This suggests the potential for *in vivo* imaging applications (Fig. 4A, B).

The results show that EcN-Rluc series strains, except for EcN-Rluc, exhibit high bioluminescence intensity when reacting with CTZ h. Under the action of CTZ B5, the luminescence process is long and stable. The EcN-Rluc8.6-CTZ and EcN-Rluc8.6-CTZ h pairs were characterized by a red-shifted spectrum. As expected, these combinations also performed excellently in *in vivo* imaging (Figs. 9, 13). Our findings confirm that Rluc8.6 has outstanding bioluminescent properties, especially in the presence of CTZ h. According to the crystal structure (Fig. 1), the C6 substituent of the CTZ skeleton, particularly the -OH group of the C6-(p-hydroxyphenyl) substituent, plays a crucial role in the interaction with the Rluc series luciferase. Meanwhile, the -OH group at the C2 position is less important for protein interactions.

### 3.3 Bioluminescence properties of engineered EcN strains containing Nluc series luciferases

We utilized the engineered EcN strain containing an Nluc luciferase series, including Nluc, teNluc, Qluc, and Antares with FRZ, to evaluate bioluminescence activity. At the beginning of the reaction, we measured the luminescence intensity after reacting different luciferases with different concentrations of FRZ substrates to analyze the luminescence intensity of the same luciferase under different substrate concentrations and different luciferases at the same substrate concentration.

**Fig. 5:**
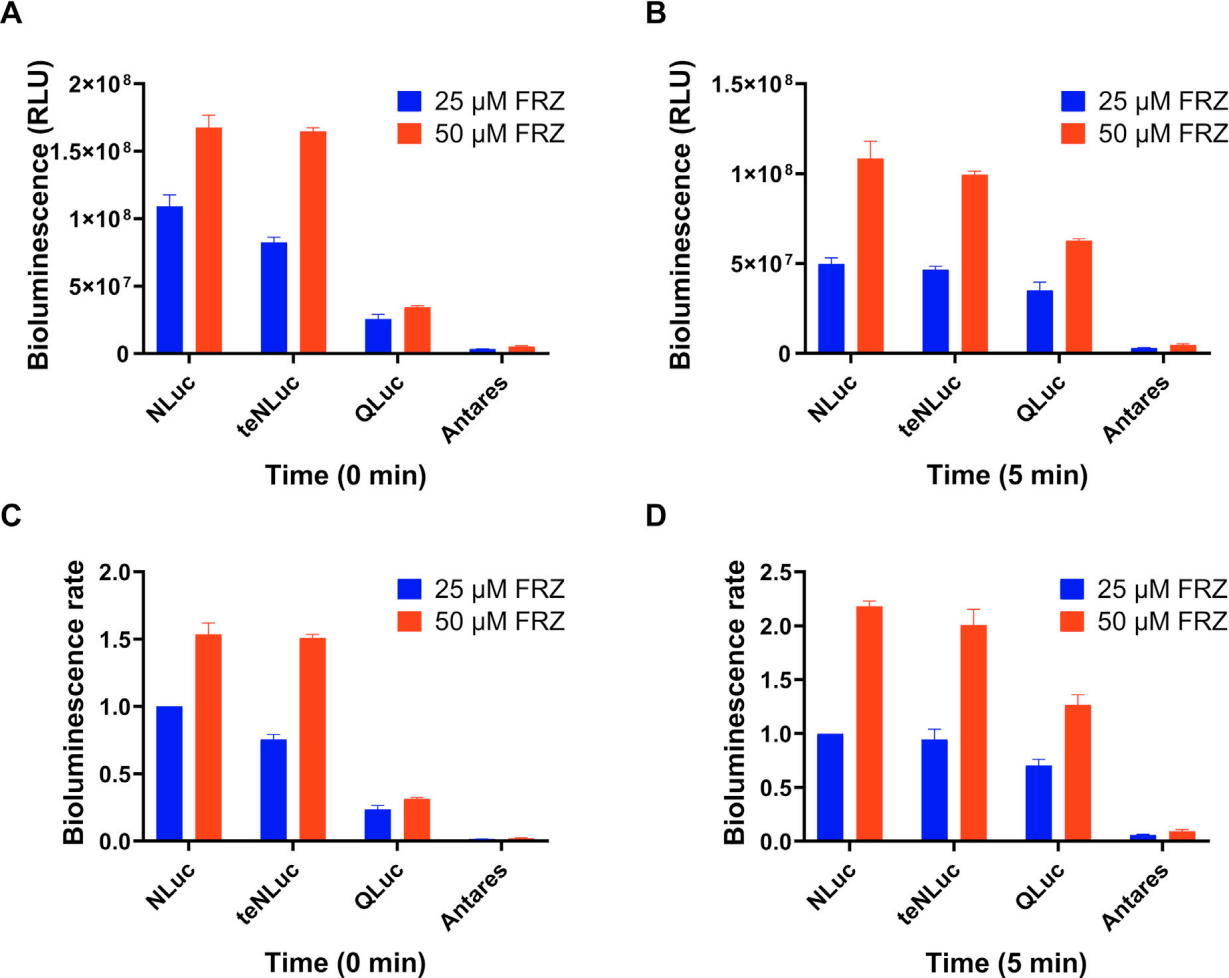
**Bioluminescence intensity of engineered EcN strains containing Nluc Series luciferases.** Bioluminescence intensity of the Nluc series EcN strains with FRZ at 0 min (A) and 5 min (B). Bioluminescence rate of the Nluc series EcN strains with FRZ (Nluc + 25 µM FRZ set to 1) at 0 min (C) and 5 min (D). The assay was performed in triplicate and is represented as the mean ± standard error of the mean (SEM).

The results suggest that the concentration of substrates is directly proportional to bioluminescence when using the same luciferase. We observed an approximate fold relationship when comparing the luminescence intensity of the same luciferase at 25 and 50 µM. However, the increase in substrate concentration was not proportional to the bioluminescence intensity, which may be due to the limited amount of luciferase. Briefly, EcN—Nluc and EcN-teNluc showed ideal reactions, with FRZ representing a greater luminescence intensity, while the luminescence intensity of EcN—Nluc decreased more rapidly. In addition, we confirmed that EcN-Qluc exhibited a gradual increase in luminescence intensity within the first 5 min (Fig. 5).

**Fig. 6:**
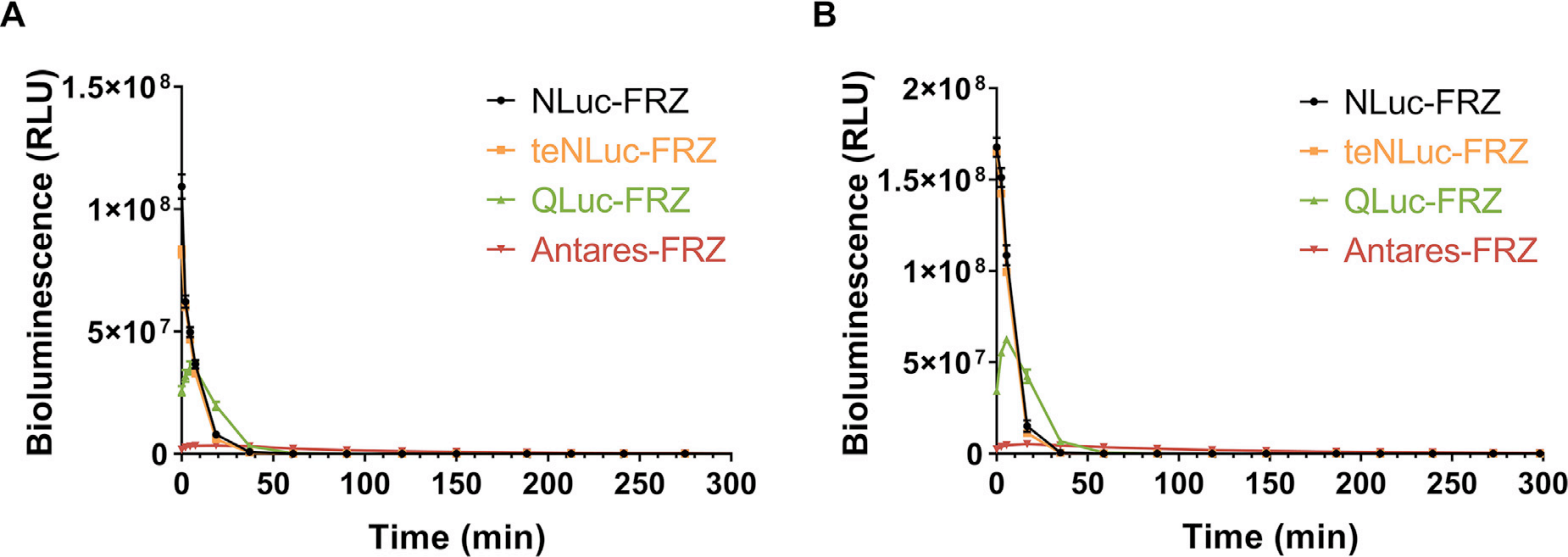
**The bioluminescence persistence of engineered EcN strains containing Nluc Series luciferases.** Bioluminescence time course of the engineered EcN strains containing Nluc series luciferases in the presence of FRZ with a final concentration of 25 µM **(**A) or 50 µM (B). The assay was performed in triplicate and is represented as the mean ± standard error of the mean (SEM).

Subsequently, we observed that the luminescence intensity followed the order EcN—Nluc, EcN-teNluc, EcN-Qluc, and EcN-Antares, with the intensity of 50 µM FRZ being approximately 1.5 times higher than that of 25 µM FRZ. Notably, the reaction between EcN-Qluc and FRZ first increased and then decreased over time, with the maximum luminescence intensity occurring at approximately 5 min, regardless of the FRZ concentration. EcN-Antares showed a slight increase followed by a decrease, reaching peak intensity at approximately 17 min and then stabilizing at a low level at 170 min. Although the luminescence intensity of both EcN—Nluc and EcN-teNluc rapidly decreased as the reaction progressed, it stabilized at a low level after 17 min (Fig. 6).

**Fig. 7:**
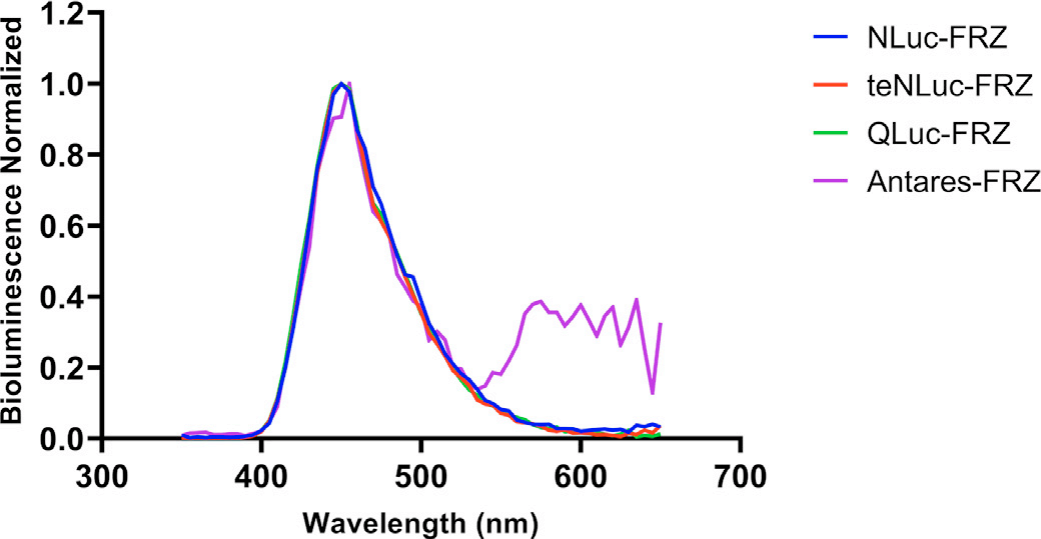
Bioluminescence emission spectra of the engineered EcN strains containing Nluc series luciferases under the action of FRZ.

The maximum light intensity was normalized to 1 for plotting the spectra to measure the bioluminescence spectra of the EcN—Nluc strains combined with their corresponding substrates. The maximum emission wavelengths of the EcN—Nluc series strains with the action of FRZ were approximately 450 nm (Fig. 7).

The results showed that the bioluminescence emission generated from the EcN—Nluc-FRZ and EcN-teNluc-FRZ pairs was strong, suggesting that the two combinations have the potential to “illuminate” themselves *in vivo* (Figs. 10, 14). In the case of Qluc-FRZ, it did not exhibit good bioluminescence properties, possibly because FRZ is not a suitable substrate [21]. Although teNluc is a mutant that evolved for another substrate [22], the EcN expressing teNluc exhibits good bioluminescence in the presence of FRZ. These results indicate that teNluc has greater tolerance to the substrate than Qluc. Additionally, the C8 substituent of its skeleton interacts with the mutant of Nluc, which is different from that of the wild-type Nluc. Antares, a bioluminescence resonance energy transfer (BRET)-like protein that fuses Nluc luciferase and CyOFP1 fluorescent protein, shows BRET performance on the spectrum, but its bioluminescence is low in EcN. Therefore, it was not chosen for *in vivo* imaging.

### 3.4 In vivo imaging of engineered bioluminescent EcN strains

After evaluating bioluminescence activity, some combinations with strong bioluminescence were selected for *in vivo* imaging studies in live animals. A xenograft mouse model was established, and recombinant strains were administered via tail vein injection. Bioluminescence was triggered by the substrate solution from intratumoral injection to assess the imaging effect at the tumor sites in the model mice.

**Fig. 8:**
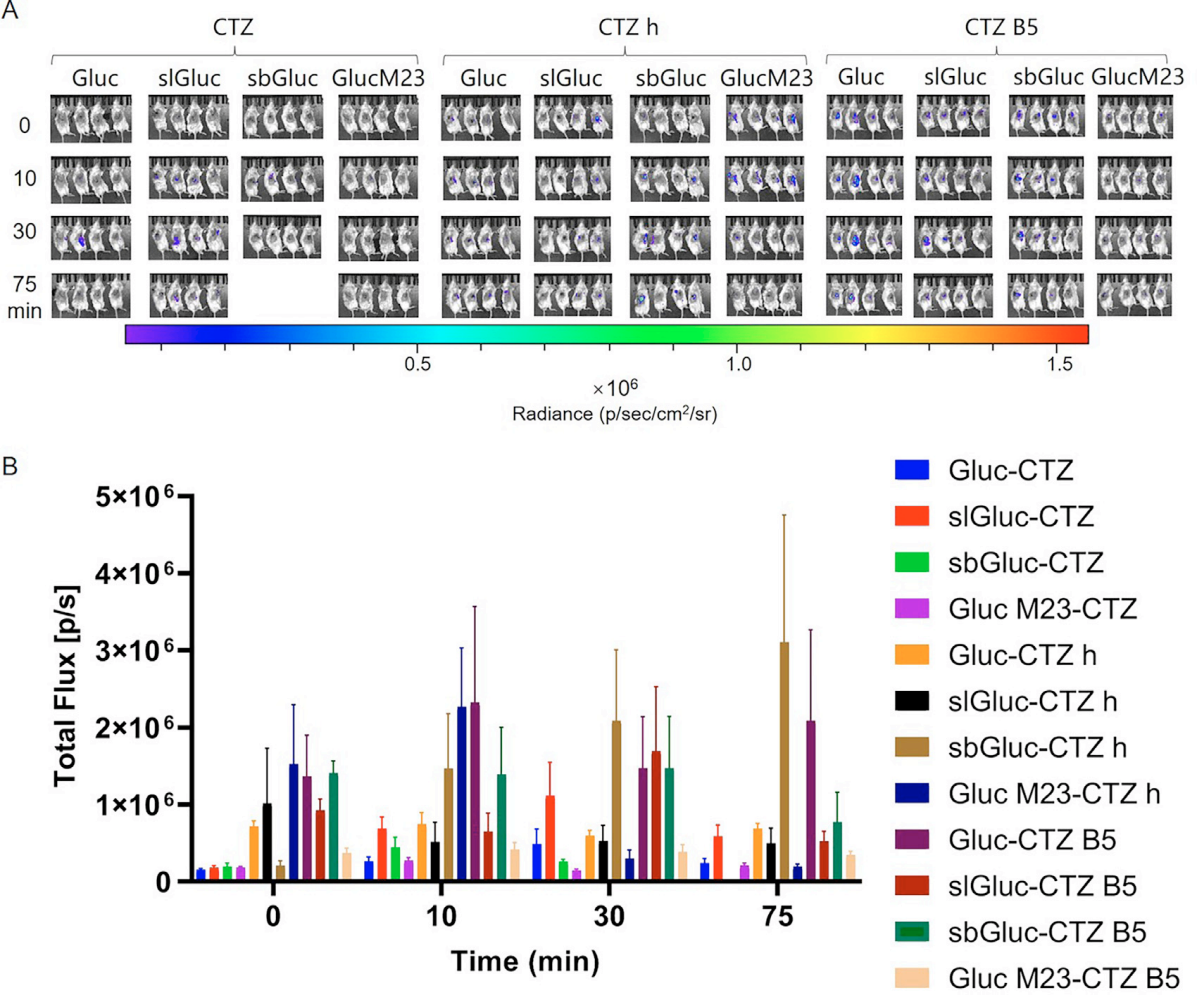
***In vivo* imaging of tumor-targeting EcN-Gluc series strains.** Mice were injected with 100 µL of EcN strains containing Gluc series luminescent elements at OD_600_ = 0.2 via the tail vein. After 48 h, 40 µL of 500 µM solutions of compounds CTZ, CTZ h, and CTZ B5 were injected directly into the tumor, respectively. Imaging was performed immediately after compounds injection, and then repeated at 10, 30, and 75 min after the injection (A). Furthermore, we measured the total flux in the tumor sites of all groups of mice at each time point to quantify the luminescence intensity therein (B). The assay was performed in triplicate and is represented as the mean ± standard error of the mean (SEM).

In the Gluc group, the combinations of EcN-sbGluc-CTZ h, EcN-Gluc-CTZ B5, EcN-slGluc-CTZ B5, and EcN-sbGluc-CTZ B5 had better imaging effects, while the rest showed relatively weak effects. Among them, the luminescence intensities of EcN-sbGluc-CTZ h and EcN-Gluc-CTZ B5 were stronger and increased over time. The luminescence intensities of EcN-slGluc-CTZ B5 and EcN-sbGluc-CTZ B5 decreased gradually throughout the measurement period and then remained relatively stable. Conversely, EcN-Gluc M23-CTZ h showed strong luminescence at the beginning of the reaction; this decreased rapidly after 10 min and was unstable (Fig. 8).

**Fig. 9:**
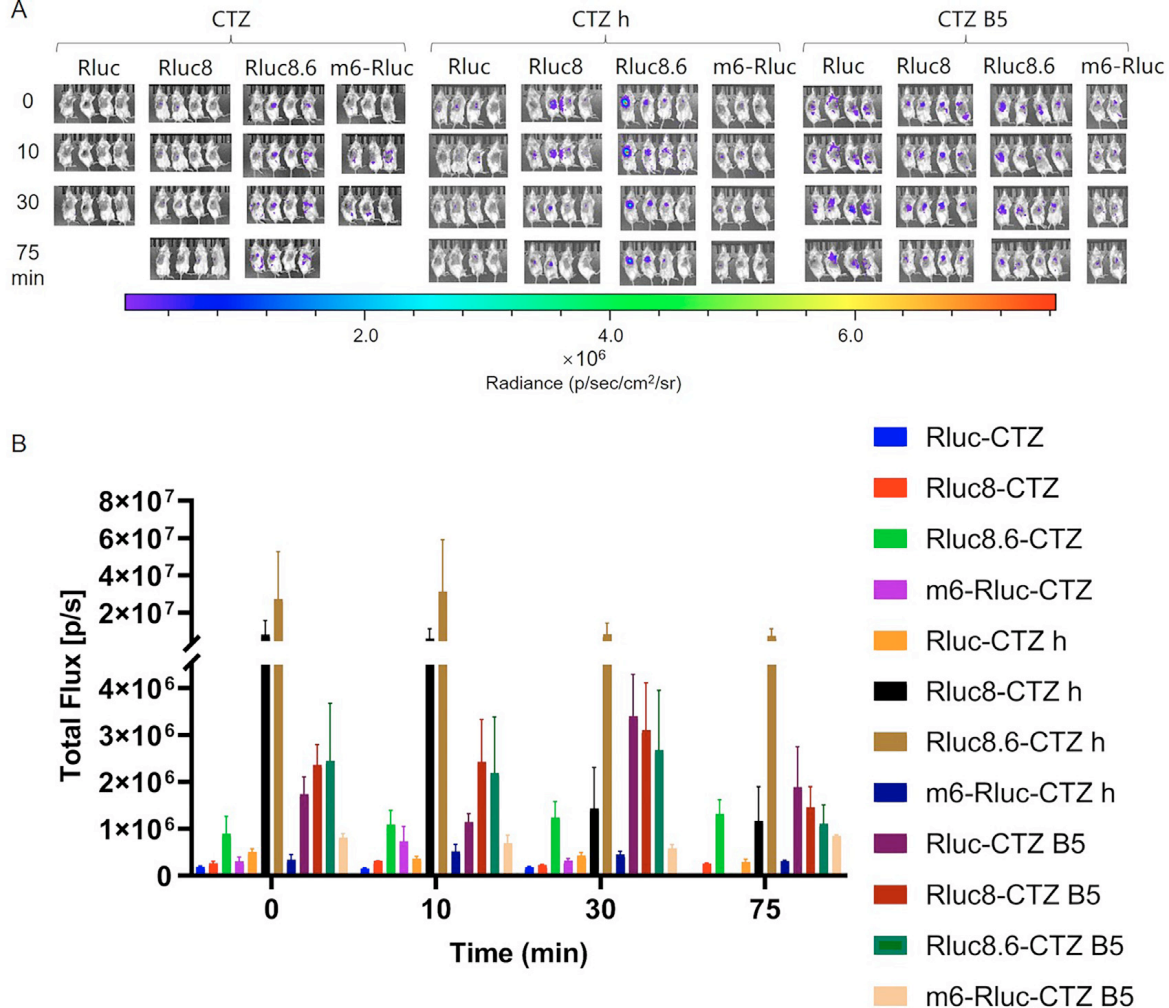
***In vivo* imaging of tumor-targeting EcN-Rluc series strains.** Mice were injected with 100 µL of EcN strains containing Rluc series luminescent elements at OD_600_ = 0.2 via the tail vein. After 48 h, 40 µL of 500 µM solutions of compounds CTZ, CTZ h, and CTZ B5 were injected directly into the tumor, respectively. Imaging was performed immediately after compounds injection, and then repeated at 10, 30, and 75 min after the injection (A). Furthermore, we measured the total flux in the tumor sites of all groups of mice at each time point to quantify the luminescence intensity therein (B). The assay was performed in triplicate and is represented as the mean ± standard error of the mean (SEM).

The combination of CTZ h and EcN-Rluc8.6 exhibited the most significant imaging effect, with a high luminescence intensity that decreased slowly over time. Its superior luminescence and red-shifted bioluminescence may be responsible for this. Among the other combinations, EcN-Rluc 8.6-CTZ, EcN-Rluc-CTZ B5, EcN-Rluc 8-CTZ B5, and EcN-Rluc 8.6-CTZ B5 indicated good luminescence with relatively stable intensity within the measurement period. The remaining combinations produced comparatively weaker imaging effects (Fig. 9).

**Fig. 10:**
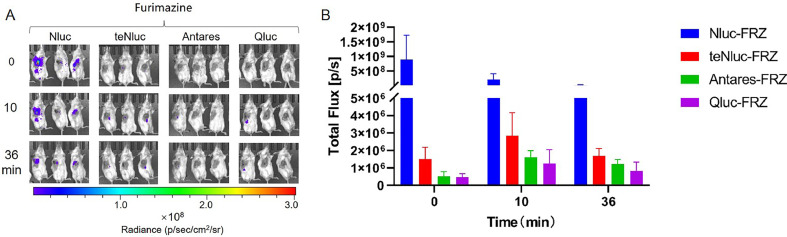
***In vivo* imaging of tumor-targeting EcN—Nluc series strains.** Mice were injected with 100 µL of EcN strains containing Nluc series luminescent elements at OD_600_ = 0.2 via the tail vein. After 48 h, 40 µL of 500 µM solutions of compound FRZ was injected directly into the tumor, respectively. Imaging was performed immediately after compounds injection, and then repeated at 10, 30 min after the injection (A). Furthermore, we measured the total flux in the tumor sites of all groups of mice at each time point to quantify the luminescence intensity therein (B). The assay was performed in triplicate and is represented as the mean ± standard error of the mean (SEM).

The imaging effect was the most significant in the EcN—Nluc group, with luminescence intensity higher than that in other groups and gradually decreasing. The other groups showed relatively weaker imaging effects, with the luminescence intensity first increasing and then decreasing (Fig. 10).

**Fig. 11:**
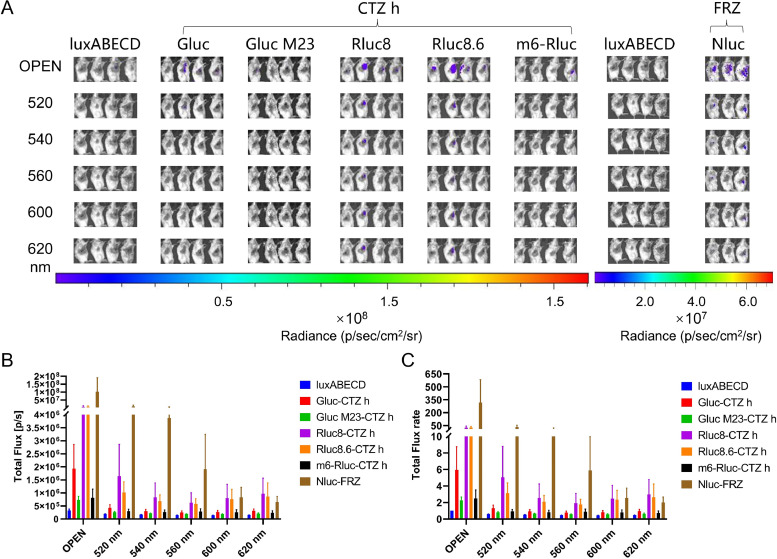
***In vivo* imaging of tumor-targeting EcN strains using different wavelength filters.** Mice were injected with 100 µL of EcN strains containing different series luminescent elements at OD_600_ = 0.2 via the tail vein. After 48 h, 40 µL of 500 µM solutions of compounds CTZ h or FRZ was injected directly into the tumor, respectively. Imaging was performed immediately after compounds injection using different filters. (A). Furthermore, we measured the total flux in the tumor sites of all groups of mice under the action of different filters to quantify the luminescence intensity therein (B). All intensity values then are normalized to LuxCDABE-OPEN (C). The assay was performed in triplicate and is represented as the mean ± standard error of the mean (SEM). The “OPEN” designation pertains to the aggregation of bioluminescence over the entire wavelength spectrum. Specifically, 520 nm, 540 nm, 560 nm, 600 nm, and 620 nm denote the utilization of optical filters with the corresponding wavelengths. In this setup, only bioluminescence with wavelengths exceeding the specified values will be collected.

To investigate the influence of red-shift wavelength on *in vivo* imaging, we handpicked six bioluminescent EcN strains that demonstrated relatively excellent imaging effects when the corresponding substrates were in place. These groups, along with EcN strain capable of expressing *luxCDABE*, were subjected to *in vivo* imaging using different wavelength filters (Fig. 11). The results revealed that, regardless of whether the overall bioluminescence intensity or the bioluminescence intensity obtained with red-shifted wavelength filters was considered, our engineered bioluminescent EcN groups surpassed the EcN-LuxCDABE group. Furthermore, EcN-Rluc8-CTZ h and EcN-Rluc8.6-CTZ h, which demonstrated a red shift *in vitro*, continued to yield excellent outcomes when imaged using high-wavelength filters. These findings suggest that engineered bioluminescent EcN groups such as EcN-Rluc8-CTZ h and EcN-Rluc8.6-CTZ h possess red-shifted wavelengths. This implies that they are better suited for *in vivo* imaging applications.

We also measured the fluorescent imaging of EcN strains that expressing fluorescent protein RFP or EGFP (Fig. S5). The fluorescent imaging of tumor regions was unclear, and the background was high. This might be because the hair of the mice generated a substantial amount of background light under the excitation light, severely affecting the final observation. In contrast, bioluminescence imaging has a low background, enabling clear imaging of tumor regions.

Subsequently, we examined the tumor colonization of engineered EcN 48 h after injection via the tail vein. The results indicate the presence of a certain number of engineered EcN capable of growing on LB agar plates containing corresponding antibiotics within the tumors (Fig. S6). Most engineered EcN strains had a tumor colonization ability ranging from approximately 1 × 10^7^ to 3 × 10^8^ at 48 h. EcN-sbGluc could achieve a colonization ability of 1 × 10^9^, while EcN-Gluc had a value of 2 × 10^6^. In comparison, the colonization effects of EcN-Rluc 8.6, EcN-m6-Rluc, EcN-teNluc, EcN-Antares, and EcN-Qluc were relatively poor. This might be due to the inhibition of the corresponding proteins on the growth of the strains or the loss of plasmids, which requires further verification in subsequent studies. Moreover, the *in vivo* imaging effects of recombinant strains with poor colonization effects, except for EcN-Rluc 8.6, were all poor, indicating a possible positive correlation between the colonization effect of the strains and the effect of *in vivo* imaging. Meanwhile, the effect exhibited by EcN-Rluc 8.6 indicates the importance of the red-shift for *in vivo* imaging.

In summary, the combination of EcN—Nluc-FRZ and EcN-Rluc 8.6-CTZ h revealed the most significant tumor imaging effect, with the luminescence intensity maintained at 10^8^–10^9^ and 10^7^, gradually decreasing over time. The luminescence intensities of EcN-sbGluc-CTZ h, EcN-Gluc-CTZ B5, EcN-Rluc 8.6-CTZ, EcN-Rluc-CTZ B5, and EcN-Rluc 8-CTZ B5 were moderate and stable (Fig. 8, Fig. 9, Fig. 10). Bioluminescence imaging with red-shifted filters indicated that some of them, especially EcN-Rluc 8.6-CTZ h and EcN-Rluc 8-CTZ h, showed red-shifted *in vivo* bioluminescence (Fig. 11). The reason the EcN-Rluc 8.6-CTZ h behaved better might be that this pair exhibits a wide red-shifted emission spectra and a strong bioluminescence, which is beneficial for *in vivo* imaging. Additionally, the CTZ-h substrate is more soluble than others, benefiting *in vivo* imaging. The Nluc-FRZ pair is one of the strongest bioluminescence pairs. Its high bioluminescence intensity enables it to perform better in *in vivo* imaging. For *in vivo* imaging, except for brighter bioluminescence, a red-shifted emission is favored [15]. Blue wavelengths are strongly attenuated in biological tissues due to hemoglobin absorption. In contrast, red wavelengths avoid hemoglobin absorption and have longer mean free paths, enabling deeper tissue imaging. Therefore, tumor-targeting bacteria labeled with red-shifted luciferase can be more easily detected in deep tissues, such as tumors.

### 3.5 In vivo imaging of gavaged engineered bioluminescent EcN strains in mouse intestinal tract

As the EcN given in this study is a probiotic chassis strain, we investigated its potential for intestinal imaging. The strains were administered via gavage in live mice, and the substrate solution was delivered by intraperitoneal injection.

**Fig. 12:**
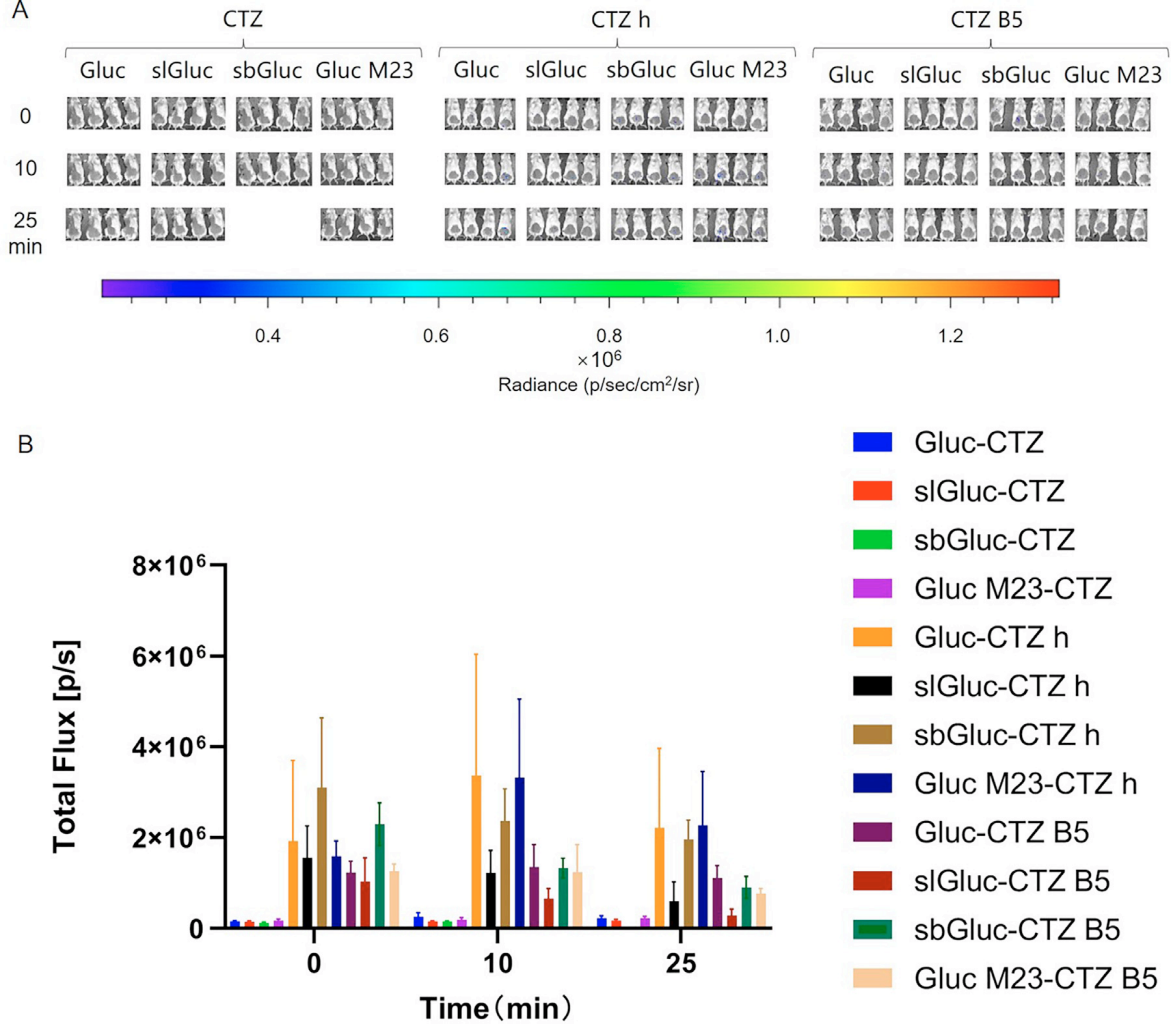
***In vivo* intestinal imaging of EcN-Gluc series strains.** Mice were gavaged with 400 µL of EcN strains containing Gluc series luminescent elements at OD_600_ = 0.8. After 1.5 h, 100 µL of 250 µM solutions of compounds CTZ were injected intraperitoneally, respectively. Imaging was performed immediately after compounds injection, then repeated 10, 25 min after the injection (A). Furthermore, we measured the total flux in the intestinal tracts of all groups of mice at each time point to quantify the luminescence intensity therein (B). The assay was performed in triplicate and is represented as the mean ± standard error of the mean (SEM).

In the EcN-Gluc series, the imaging effects were similar across other combinations except for the CTZ group, which showed a weak luminescence intensity. Specifically, the EcN-sbGluc groups displayed a gradual decrease in luminescence, and the imaging intensity in the EcN-Gluc group increased initially and then decreased over time, reaching its peak at approximately 10 min. In contrast, the EcN-Gluc M23 groups showed a gradual increase in luminescence intensity until approximately 25 min (Fig. 12).

**Fig. 13:**
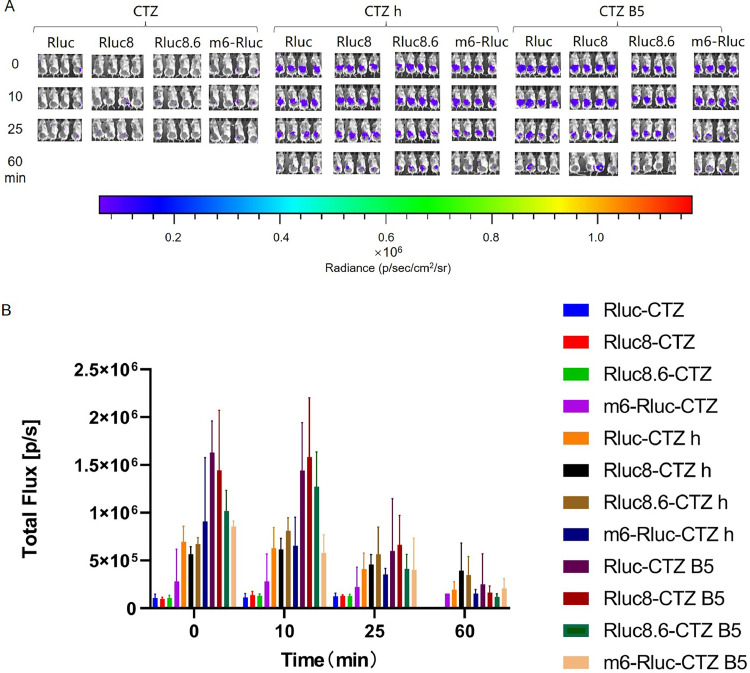
***In vivo* intestinal imaging of EcN-Rluc series strains.** Mice were gavaged with 400 µL of EcN strains containing Rluc series luminescent elements at OD_600_ = 0.8. After 1.5 h, 100 µL of 250 µM solutions of compounds CTZ, CTZ h, and CTZ B5 were injected intraperitoneally, respectively. Imaging was performed immediately after compounds injection, then repeated 10, 25, and 60 min after the injection (A). Furthermore, we measured the total flux in the intestinal tracts of all groups of mice at each time point to quantify the luminescence intensity therein (B). The assay was performed in triplicate and is represented as the mean ± standard error of the mean (SEM).

In the EcN-Rluc series, the luminescence intensity of the CTZ group remained very low. The CTZ B5 group showed better effects compared to CTZ h. Among the combinations, EcN-Rluc-CTZ B5, EcN-Rluc 8-CTZ B5, and EcN-Rluc 8.6-CTZ B5 provided the best performance, with relatively high luminescence intensities. However, they then experienced a rapid decline in luminescence intensity after a short period of increase (Fig. 13).

**Fig. 14:**
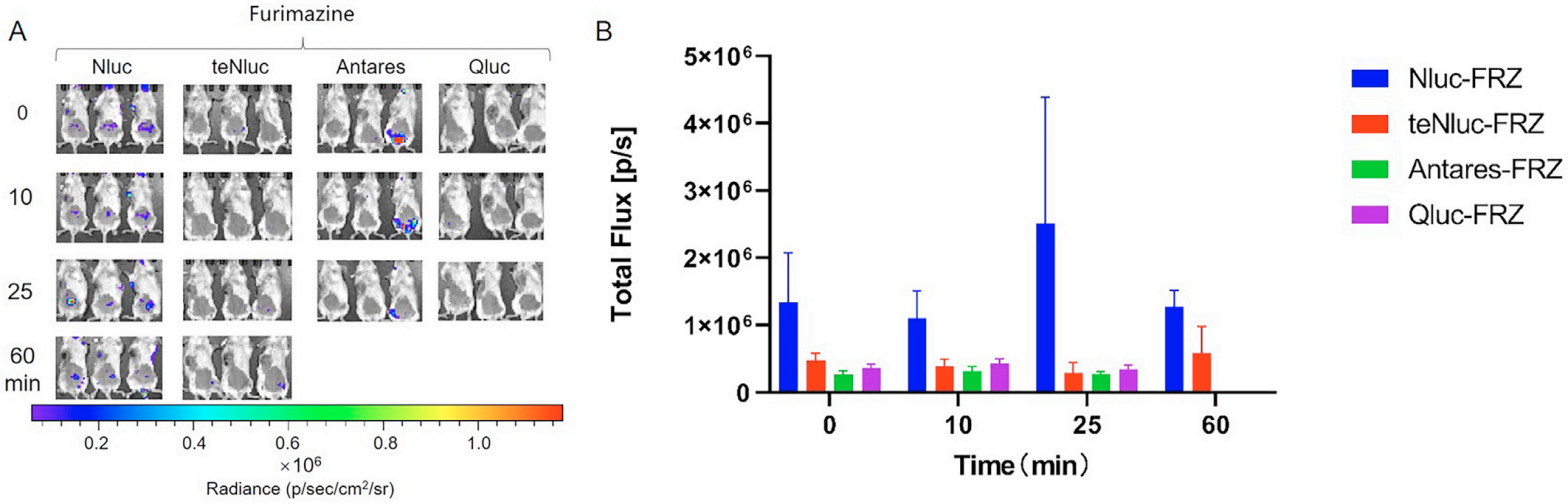
***In vivo* intestinal imaging of EcN—Nluc series strains.** Mice were gavaged with 400 µL of EcN strains containing Nluc series luminescent elements at OD_600_ = 0.8. After 1.5 h, 100 µL of 250 µM solutions of compounds FRZ were injected intraperitoneally, respectively. Imaging was performed immediately after FRZ injection, then repeated at 10, 25, and 60 min after the injection (A). Furthermore, we measured the total flux in the intestinal tracts of all groups of mice at each time point to quantify the luminescence intensity therein (B). The assay was performed in triplicate and is represented as the mean ± standard error of the mean (SEM).

In the EcN—Nluc series, the EcN—Nluc-FRZ pair exhibited the most significant imaging effect, with high luminescence intensity that initially increased and then decreased over time, peaking at approximately 25 min. The other groups displayed relatively weaker imaging effects; however, all the groups showed a similar trend of increasing intensity followed by a decrease (Fig. 14).

Overall, the recombinant EcN strains demonstrated promising effects in intestinal imaging, with luminescence intensities generally reaching 10^6^. The combinations of EcN-Gluc-CTZ h, EcN-sbGluc-CTZ h, EcN-Gluc M23-CTZ h, and EcN—Nluc-FRZ revealed the best imaging effects, maintaining stable luminescence above 10^6^ for a long period. EcN-Rluc-CTZ B5 and EcN-Rluc 8-CTZ B5 showed good luminescence at the start of the reaction but rapidly declined after 10 min (Fig. 12, Fig. 13, Fig. 14). The combination of the EcN-Rluc series and CTZ-type substrates and the EcN—Nluc-FRZ group appear to be suitable for intestinal imaging, similar to the *in vivo* imaging of tumors.

This study shows that bright, continuous, and especially better red-shifted signals are the key conditions for the development of bioluminescence imaging tools for tumor-targeting bacteria. Furthermore, in this study, we successfully constructed 12 types of EcN recombinant strains that express distinct luciferases using gene-editing technology. Through combination screening and other relevant approaches, we examined the *in vitro* bioluminescence effects of 36 combinations, along with the bioluminescence effects within tumors and intestines for 28 combinations (Table 3). Among these, several combinations demonstrated relatively high bioluminescence intensity, a stable luminescence process, or achieved a red shift in luminescence. In the subsequent steps, we can explore strategies like BRET to enhance the efficacy of bioluminescence imaging [7]. The recombinant EcN strains and the corresponding test results lay a solid foundation and offer a valuable reference for the future development of bioluminescence tools for tumor-targeting bacteria.

**Table 3:** Bioluminescence properties of various luciferase-expressing EcN-luciferin pairs in our study.

		Relative intensity		
	Peak of wavelength (nm) a	EcN cells b	Mice	
			EcN cells in tumor c	EcN cells in intestinal tract^d^
		Relative intensity	Relative intensity	Relative intensity
Rluc-CTZ	490	1	1	1
Rluc8-CTZ	485	2.47	1.68	1
Rluc8.6-CTZ	535	0.07	5.92	0.95
m6-Rluc-CTZ	490	0.02	3.99	2.04
Rluc-CTZ h	490	13.16	1.96	4.57
Rluc8-CTZ h	485	47.16	33.42	4.47
Rluc8.6-CTZ h	530	1.25	170.61	5.89
m6-Rluc-CTZ h	485	0.46	2.81	4.76
Rluc-CTZ B5	415	5.26	6.22	10.49
Rluc8-CTZ B5	415	15.17	13.22	11.51
Rluc8.6-CTZ B5	415	2.1	11.92	9.25
m6-Rluc-CTZ B5	420	0.2	3.77	4.21
Rluc-CTZ B12	400	6.12	N.D.	N.D.
Rluc8-CTZ B12	410	17.19	N.D.	N.D.
Rluc8.6-CTZ B12	400	0.56	N.D.	N.D.
m6-Rluc-CTZ B12	405	0.14	N.D.	N.D.
Gluc-CTZ	N.D.	1	1	1
slGluc-CTZ	N.D.	1.09	4.46	0.6
sbGluc-CTZ	N.D.	0.98	2.9	0.58
GlucM23-CTZ	N.D.	0.95	1.78	0.75
Gluc-CTZ h	N.D.	1.07	4.84	13.23
slGluc-CTZ h	N.D.	0.95	3.34	4.81
sbGluc-CTZ h	N.D.	1	9.51	9.32
GlucM23-CTZ h	N.D.	1.25	14.7	13.08
Gluc-CTZ B5	N.D.	1.8	15.06	5.32
slGluc-CTZ B5	N.D.	1.64	4.21	2.57
sbGluc-CTZ B5	N.D.	1.93	9	5.22
GlucM23-CTZ B5	N.D.	1.84	2.68	4.87
Gluc-CTZ B12	N.D.	0.52	N.D.	N.D.
slGluc-CTZ B12	N.D.	0.45	N.D.	N.D.
sbGluc-CTZ B12	N.D.	0.44	N.D.	N.D.
GlucM23-CTZ B12	N.D.	0.47	N.D.	N.D.
Nluc-FRZ	450	1	1	1
teNluc-FRZ	450	0.76	0.013	0.36
Qluc-FRZ	450	0.24	0.006	0.39
Antares-FRZ	455/575	0.02	0.008	0.29

a The maximum emission wavelengths of the engineered EcN strains with the action of different substrates.

b Bioluminescence rate of the engineered EcN strains with 25 µM CTZ analogs or FRZ (Rluc + CTZ, Gluc + CTZ, and Nluc + FRZ set to 1 for each series) at 0 min.

c Bioluminescence rate at the tumor site of the engineered EcN strains 10 min after intratumoral injection of 40 µL 500 µM CTZ analogs or FRZ (Rluc + CTZ, Gluc + CTZ, and Nluc + FRZ set to 1 for each series). N.D., Not detected.

d Bioluminescence rate in the intestinal tracts of the engineered EcN strains 10 min after intraperitoneal injection of 100 µL 250 µM CTZ analogs or FRZ (Rluc + CTZ, Gluc + CTZ, and Nluc + FRZ set to 1 for each series). N.D., Not detected.

## 4 Conclusion

This study provides diverse and efficient bioluminescent imaging tools for bacteria-mediated tumor therapy research. We used the tumor-targeting bacteria, EcN, as the host to engineer three series of bioluminescent strains based on the principles of CTZ-type bioluminescence, offering various bacterial imaging and tracing modes. We evaluated the bioluminescence properties of these systems, including bioluminescence intensity, time-course, spectra, and *in vivo* imaging. The results demonstrated that different combinations of luciferase mutants and CTZ analogs produced diverse bioluminescent properties. Among them, we found that multiple combinations showed powerful, red-shifted, and long-term bioluminescence. EcN-Rluc8 strain showed stable and high luminescence *in vivo* with various substrates. The combination of the EcN-Rluc8.6 strain and CTZ h has been confirmed to show advantages for *in vivo* imaging, as it has a maximum emission wavelength of 530 nm, featuring a red-shifted spectrum with strong and stable luminescence intensity. The bioluminescence duration of the EcN-Rluc8.6-CTZ B5 pair was the best. The EcN-m6-Rluc strain exhibited better bioluminescence intensity and persistence under the action of CTZ and CTZ h. Because the luciferase mutant m6-Rluc has high thermal stability, it is expected to have great development value. The EcN-Gluc-CTZ h pair and EcN-Gluc M23-CTZ h pair performed the best among the combination of CTZ analogs and EcN-Gluc series strains, particularly in intestinal imaging. Regarding the combination of FRZ and the EcN—Nluc series, the EcN—Nluc-FRZ pair and the EcN-teNluc-FRZ pair showed exceptionally high luminescence intensity *in vitro*. Moreover, the EcN—Nluc-FRZ pair also showed remarkable luminescence intensity and stability during *in vivo* imaging.

By comparing the bioluminescence properties of the engineered EcN strains with the *in vivo* imaging results, we found that the bioluminescence intensity, bioluminescence persistence, and maximum emission wavelengths exhibited by the engineered EcN strains *in vitro* affected their final *in vivo* imaging effects. Excluding the EcN-m6-Rluc, EcN-teNluc, EcN-Antares, and EcN-Qluc groups, which were affected by poor colonization effects, the EcN-Rluc8-CTZ h and EcN—Nluc-FRZ groups, which showed significant bioluminescence intensity in the *in vitro* detection, demonstrated excellent performance in *in vivo* imaging. Meanwhile, EcN-Rluc8.6-CTZ and EcN-Rluc8.6-CTZ h with red-shifted properties, although not having significant bioluminescence intensity, stood out under the action of the same substrate and showed better *in vivo* imaging effects.

Bacterial imaging and tracing remain challenging, especially under *in vivo* conditions. This study explored the design and development of bacterial imaging tools, providing valuable insights into the optimization of multiple visualization systems suitable for *in vivo* imaging of tumors and the intestines. Future development requires the utilization of diversified strategies to establish more efficient bacterial imaging and tracing tools to monitor real-time bacterial growth, reproduction, and distribution *in vivo*. Further exploration of bacterial tumor-targeting tendency and anti-tumor potential is required to provide novel methods for tumor therapy.

## Data Availability Statement

All data needed to evaluate the conclusions in the paper are present in the paper and/or the Supplementary Materials.

## CRediT authorship contribution statement

**Chenghao Ma:** Writing – original draft, Methodology, Data curation. **Jingxi Liu:** Methodology. **Hongjing Liu:** Methodology, Data curation. **Xiaohan Zhao:** Methodology, Data curation. **Geng Li:** Methodology. **Youming Zhang:** Writing – review & editing, Supervision. **Tianyu Jiang:** Writing – review & editing, Supervision, Conceptualization.

## Declaration of Competing Interest

The authors declare the following financial interests/personal relationships which may be considered as potential competing interests: Given his role as Editor-in-Chief, Dr. Youming Zhang, had no involvement in the peer-review of this article and has no access to information regarding its peer-review. Full responsibility for the editorial process for this article was delegated to Dr. Dongchun Ni.

## Appendix Supplementary materials

Image, application 1
