# Supplementary material for: Tumor-targeting bioluminescent bacteria for in vivo imaging
Source: Eng Microbiol. 2025 Jul 11;5(3):100224. doi: 10.1016/j.engmic.2025.100224 (PMC12967832; doi:10.1016/j.engmic.2025.100224)
Supplement: Supplementary file 1 [file mmc1.docx]

Supporting Information

**Tumor-targeting bioluminescent bacteria for *in vivo* imaging**

Chenghao Ma^1^, Jingxi Liu^2^, Hongjing Liu^3^, Xiaohan Zhao^3^, Geng Li^1^, Qi Gao^3^, Sizhe Deng^3^, Youming Zhang^1^*, and Tianyu Jiang^1^*.

^1^ State Key Laboratory of Microbial Technology, Institute of Microbial Technology, Helmholtz International Lab for Anti-Infectives, Shandong University–Helmholtz Institute of Biotechnology, Shandong University, Qingdao, 266237, China

^2^ Monash Institute of Pharmaceutical Sciences, Faculty of Pharmacy and Pharmaceutical Sciences, Monash University, Melbourne, Victoria, 3052, Australia.

*^3^ School of Life Sciences, Shandong University; Qingdao, 266237, China.*

∗ Corresponding authors

E-mail addresses: [zhangyouming@sdu.edu.cn](mailto:zhangyouming@sdu.edu.cn), [tianyujiang@sdu.edu.cn](mailto:tianyujiang@sdu.edu.cn)

Content

[Optimization of combinatorial screening conditions of Engineered EcN strains containing Gluc, Rluc and Nluc Series luciferases 2](#_Toc192751371)

[Table S1 Bacterial strains used in this study. 3](#_Toc192751372)

[Table S2 Plasmids used in this study. 4](#_Toc192751373)

[Table S3 Primers used in this study 5](#_Toc192751374)

[Figure S1. Analysis of plasmid restriction and PCR analysis of engineered EcN strains. 6](#_Toc192751375)

[Figure S2. Optimization of combinatorial screening conditions for Gluc and mutant strains. 7](#_Toc192751376)

[Figure S3. Optimization of combinatorial screening conditions for Rluc and mutant strains. 8](#_Toc192751377)

[Figure S4. Optimization of screening conditions for Nluc and mutant strain combinations. 9](#_Toc192751378)

[Figure S5. The contrast of fluorescent imaging and bioluminescence imaging 10](#_Toc192751379)

[Figure S6. The CFU of engineered EcN colonized in the tumor. 11](#_Toc192751380)

##### Optimization of combinatorial screening conditions of Engineered EcN strains containing Gluc, Rluc and Nluc Series luciferases

Take Gluc for example, firstly, we tested the impact of lysate from bacterial cultures with different OD_600_ values on luminescence to determine the suitable concentration used in the following studies. The bacterial culture was diluted to OD_600_ of 0.2, 0.5, and 0.8 concentration with the corresponding buffer solution to measure the bioluminescence. As shown in Fig. S1 A and C, the response trend of Gluc and its mutants to CTZ and DeepBlueC increased with the increase of bacterial culture OD_600_ at a substrate concentration of 25 μM, where CTZ increased from 1000 RLU to 15000 RLU, DeepBlueC increased from 1000 RLU to 10000 RLU. The results in the figure show that the OD_600_ of the experimental group with the highest luminescence intensity is 0.8. Then, we examined the effect of compound concentration on luminescence intensity. The compound was diluted in the corresponding buffer to achieve substrate concentrations of 0.5, 1, 2, 5, 10, and 25 μM, maintaining a fixed OD_600_ value of 0.8 as the baseline. We found that the response level of each Gluc mutant to CTZ and DeepBlueC was positively correlated with the substrate concentration (Fig. S2). At the same time, the optimal combination conditions of Rluc and Nluc is the same as that of Gluc (Fig. S3, S4).

##### Table S1 Bacterial strains used in this study.

| Bacterial strains | Characteristics | Source |
| --- | --- | --- |
| *E. coli* GB2005 | (HS996, ∆recET, ∆ybcC). The endogenous recET locus and the DLP12 prophage ybcC,  which encodes a putative exonuclease similar to the Redα, were deleted | Our lab (Fu, et al. 2010) |
| *E. coli* GB08-red | (GB2005, araC-BAD-γβαA) *redγβα* and *recA* under BAD promoter were inserted at the *ybcC* locus | Our lab (Fu, et al. 2010) |
| *E. coli* GB05-dir | (GB2005, araC-BAD-ETgA) recE, recT, redγ and recA under BAD promoter were inserted at the ybcC locus | Our lab (Fu, et al. 2010) |
| *E. coli* GBdir-gyrA462 | GB05-dir, GyrA mutation of Arg462Cys | Our lab (Fu, et al. 2010) |
| *E. coli* GBred-gyrA462 | GB08-red, GyrA mutation of Arg462Cys | Our lab (Fu, et al. 2010) |
| EcN | *E. coli Nissle* 1917 wild type (WT) strain | Ardeypharm |
| EcN-RFP | EcN harboring plasmid pSB1C3-lac-RFP-CmR | Our lab |
| EcN-EGFP | EcN harboring plasmid pSB1C3-lac-Fluc-EGFP-CmR | Our lab |
| EcN-luxABECD | EcN harboring plasmid pSB1C3-lac-luxABECD-CmR | Our lab |
| EcN-Gluc | EcN harboring plasmid pSB1C3-lac-Gluc-CmR | This study |
| EcN-sbGlu | EcN harboring plasmid pSB1C3-lac-sbGluc-CmR | This study |
| EcN-slGlu | EcN harboring plasmid pSB1C3-lac-slGluc-CmR | This study |
| EcN-Gluc M23 | EcN harboring plasmid pSB1C3-lac-Gluc M23-CmR | This study |
| EcN-Nluc | EcN harboring plasmid pSB1C3-lac-Nluc-CmR | This study |
| EcN-teNluc | EcN harboring plasmid pSB1C3-lac-teNluc-CmR | This study |
| EcN-Qluc | EcN harboring plasmid pSB1C3-lac-Qluc-CmR | This study |
| EcN-Antares | EcN harboring plasmid pSB1C3-lac-Antares-CmR | This study |
| EcN-Rluc | EcN harboring plasmid pSB1C3-lac-Rluc-CmR | This study |
| EcN-Rluc8 | EcN harboring plasmid pSB1C3-lac-Rluc8-CmR | This study |
| EcN-Rluc8.6 | EcN harboring plasmid pSB1C3-lac-Rluc8.6-CmR | This study |
| EcN-m6-Rluc | EcN harboring plasmid pSB1C3-lac-m6-Rluc-CmR | This study |

##### Table S2 Plasmids used in this study.

| Plasmids | Characteristics | Source |
| --- | --- | --- |
| QLuc in pUC-GW-KanR | pUC57 replicon, KanR, Qluc | Gnenwiz |
| m6-Rluc part in pUC-GW-KanR | pUC57 replicon, KanR, m6-Rluc part | Genewiz |
| pSB1C3-lac-RFP-CmR | pSB1C3 replicon, CmR, RFP | Our lab |
| pSB1C3-lac-Fluc-EGFP-CmR | pSB1C3 replicon, CmR, EGFP | Our lab |
| pSB1C3-lac-luxABECD-CmR | pSB1C3 replicon, CmR, luxABECD | Our lab |
| pSB1C3-lac-Gluc-CmR | pSB1C3 replicon, CmR, *Gaussia* Luciferase Wild type | This study |
| pSB1C3-lac-sbGluc-CmR | pSB1C3 replicon, CmR, mutate M43L, M110L, based on Gluc | This study |
| pSB1C3-lac-slGluc-CmR | pSB1C3 replicon, CmR, pSB1C3 replicon, CmR, mutate F72W, 173L, based on Gluc | This study |
| pSB1C3-lac-Gluc M23-CmR | pSB1C3 replicon, CmR, mutate K33E, M43L, V96D, M110I, G167D, based on Gluc | This study |
| pSB1C3-lac-Nluc-CmR | pSB1C3 replicon, CmR, NanoLuc Wild type | This study |
| pSB1C3-lac-teNluc-CmR | pSB1C3 replicon, CmR, mutate D19S, D85N, C164H based on Nluc | This study |
| pSB1C3-lac-Qluc-CmR | pSB1C3 replicon, CmR, mutate EP-PCR -175F, V821, P84R, A92V, based on LumiLuc | This study |
| pSB1C3-lac-Antares-CmR | pSB1C3 replicon, CmR, Antares (An optimized fusion of CyOFP1 and Nluc) | This study |
| pSB1C3-lac-Rluc-CmR | pSB1C3 replicon, CmR, *Renilla* Luciferase Wild type | This study |
| pSB1C3-lac-Rluc8-CmR | pSB1C3 replicon, CmR, mutate K136R, M253L, S287L, T2A, A55T, S130A, A143M, C124A, MI85V, based on Rluc | This study |
| pSB1C3-lac-Rluc8.6-CmR | pSB1C3 replicon, CmR, mutate T2A, ASST, A123S, C124A, S130A, K136R, A143M, D154M, E155G, D162E, I163L, M185L, M253L, L287S, based on Rluc | This study |
| pSB1C3-lac-m6-Rluc-CmR | pSB1C3 replicon, CmR, mutate F116L、I137V、I75A、N178D、N264S、S287P , based on Rluc | This study |

CmR, KanR indicate chloramphenicol, and kanamycin resistance, respectively.

##### Table S3 Primers used in this study

| Primer | Sequence (5’-3’) |
| --- | --- |
| Primer 106-vec-R | ctagtatttctcctctttctctagtatgtg |
| Primer 107-vec-F | tacccatacgatgttccagattacgcttaacgctgatagtgctagtg |
| Primer 108-Rluc-F | acaatttcacacatactagagaaagaggagaaatactagatgacttcgaaagtttatga |
| Primer 109-Rluc-R | ctatcagcgttaagcgtaatctggaacatcgtatgggtattgttcatttttgagaactc |
| Primer 110-Rluc8-F | acaatttcacacatactagagaaagaggagaaatactagatggcttccaaggtgtacga |
| Primer 111-Rluc8-R | ctatcagcgttaagcgtaatctggaacatcgtatgggtactgctcgttcttcagcacgc |
| Primer 110-Rluc8-F | acaatttcacacatactagagaaagaggagaaatactagatggcttccaaggtgtacga |
| Primer 111-Rluc8-R | ctatcagcgttaagcgtaatctggaacatcgtatgggtactgctcgttcttcagcacgc |
| Primer 112-Gluc-F | acaatttcacacatactagagaaagaggagaaatactagatgaagcccaccgagaacaa |
| Primer 113-Gluc-R | ctatcagcgttaagcgtaatctggaacatcgtatgggtagtcaccaccggcccccttga |
| Primer 112-Gluc-F | acaatttcacacatactagagaaagaggagaaatactagatgaagcccaccgagaacaa |
| Primer 113-Gluc-R | ctatcagcgttaagcgtaatctggaacatcgtatgggtagtcaccaccggcccccttga |
| Primer 112-Gluc-F | acaatttcacacatactagagaaagaggagaaatactagatgaagcccaccgagaacaa |
| Primer 113-Gluc-R | ctatcagcgttaagcgtaatctggaacatcgtatgggtagtcaccaccggcccccttga |
| Primer 112-Gluc-F | acaatttcacacatactagagaaagaggagaaatactagatgaagcccaccgagaacaa |
| Primer 117-Gluc M23-R | ctatcagcgttaagcgtaatctggaacatcgtatgggtagtcgtcaccggcccccttga |
| Primer 114-Nluc-F | acaatttcacacatactagagaaagaggagaaatactagatggtcttcacactcgaaga |
| Primer 115-Nluc-R | ctatcagcgttaagcgtaatctggaacatcgtatgggtacgccagaatgcgttcgcaca |
| Primer 114-Nluc-F | acaatttcacacatactagagaaagaggagaaatactagatggtcttcacactcgaaga |
| Primer 116-teNluc-R | ctatcagcgttaagcgtaatctggaacatcgtatgggtacgccagaatgcgttcgtgca |
| 407-for | acaatttcacacatactagagaaagaggagaaatactagatggtcttcactctcgggga |
| 408-rev | ctatcagcgttaagcgtaatctggaacatcgtatgggtacgccagaatacgttcatgca |
| 410-for | catgttgtgccacatattgagccagtagcgcggtgtattgcaccagaccttattggtat |
| 411-rev | tttgatatattttcccatttcatcaggtgcatcttcttgcggaaaatgaagacctttta |
| 412-for | caagaagatgcacctgatgaa |
| 413-rev | aatacaccgcgctactggctc |
| 422-for | tacccatacgatgttccagattacgcttaacgctgatagtgctagtgtagatcgctact |
| Primer- 414-BamHI-for | gccggatccgatggtgagcaagggcgaggagctg |
| Primer 416 rev | acgaaatcttcgagtgtgaagcctccgcccaggttggagt |
| Primer 417-for | actccaacctgggcggaggcttcacactcgaagatttcgt |
| Primer 415-HindIII-rev | cggaagcttgatcagctcgtgcctcgccagaatgcgttcgcacagc |
| Primer 418-HindIII-for | gccaagcttaacatgagaagcaagctgtacctg |
| Primer 420-rev | cgatctacactagcactatcagcgttattacttgtacagctcgtccatgc |
| Primer 421-for | gcatggacgagctgtacaagtaataacgctgatagtgctagtgtagatcg |
| Primer 419-BamHI-rev | cggggatccctagtatttctcctctttctctagtatgt |
| 410-for | catgttgtgccacatattgagccagtagcgcggtgtattgcaccagaccttattggtat |
| 411-rev | tttgatatattttcccatttcatcaggtgcatcttcttgcggaaaatgaagacctttta |
| 412-for | caagaagatgcacctgatgaa |
| 413-rev | aatacaccgcgctactggctc |


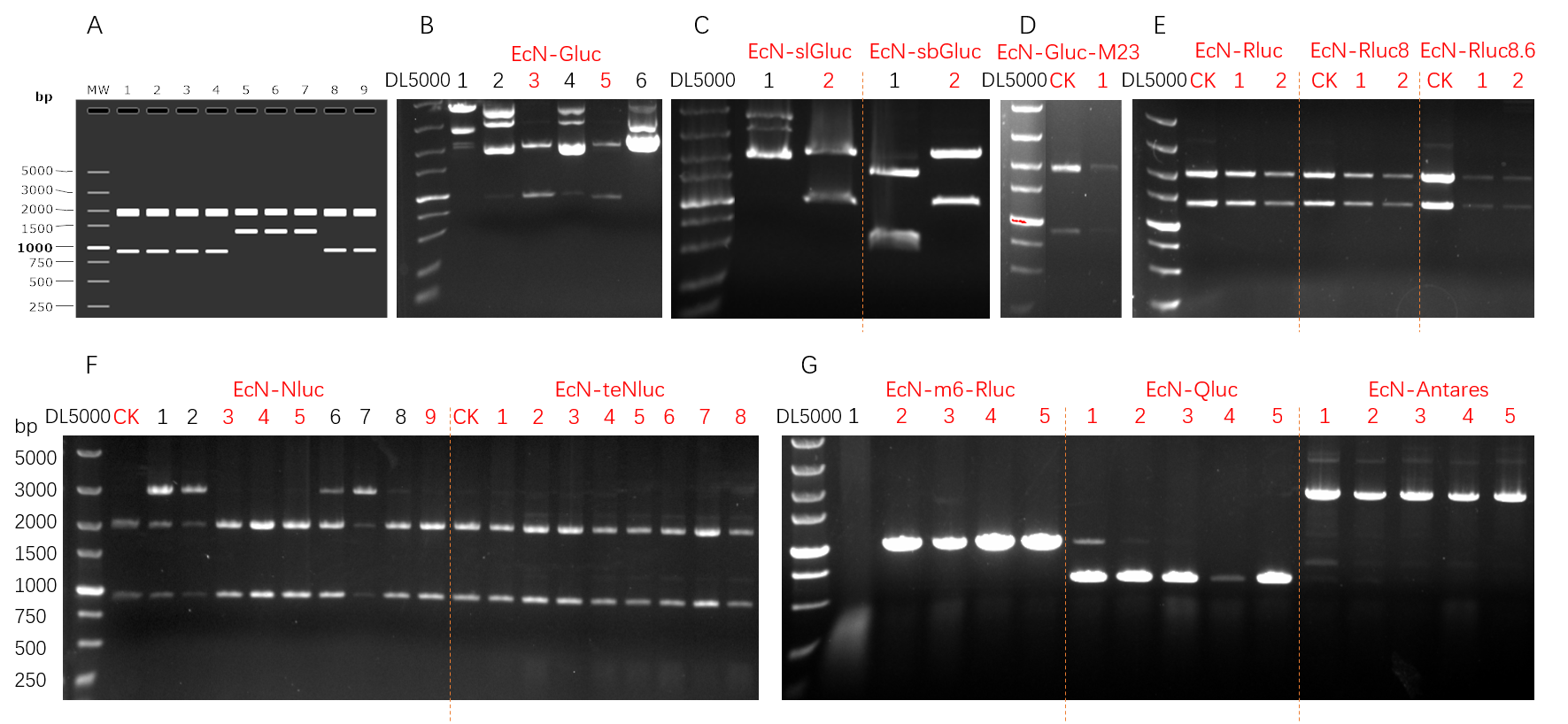


##### Figure S1. Analysis of plasmid restriction and PCR analysis of engineered EcN strains.

(A) NotI restriction analysis profiles of plasmids pSB1C3-Gluc, pSB1C3-slGluc, pSB1C3-sbGluc, pSB1C3-Gluc-M23, pSB1C3-Rluc, pSB1C3-Rluc8, pSB1C3-Rluc8.6, pSB1C3-Nluc and pSB1C3-teNluc. (B) NotI restriction analysis of plasmid pSB1C3-Gluc from EcN. (C) NotI restriction analysis of plasmid pSB1C3-slGluc or pSB1C3-sbGluc from EcN. (D) NotI restriction analysis of plasmid pSB1C3-Gluc-M23 from EcN. (E) NotI restriction analysis of plasmid pSB1C3-Rluc, pSB1C3-Rluc8 or pSB1C3-Rluc8.6 from EcN. (F) NotI restriction analysis of plasmid pSB1C3-Nluc or pSB1C3-teNluc from EcN. (G) Colony PCR analysis of m6-Rluc, Qluc or Antares fragment from the corresponding strains. Correct clones are indicated in red.


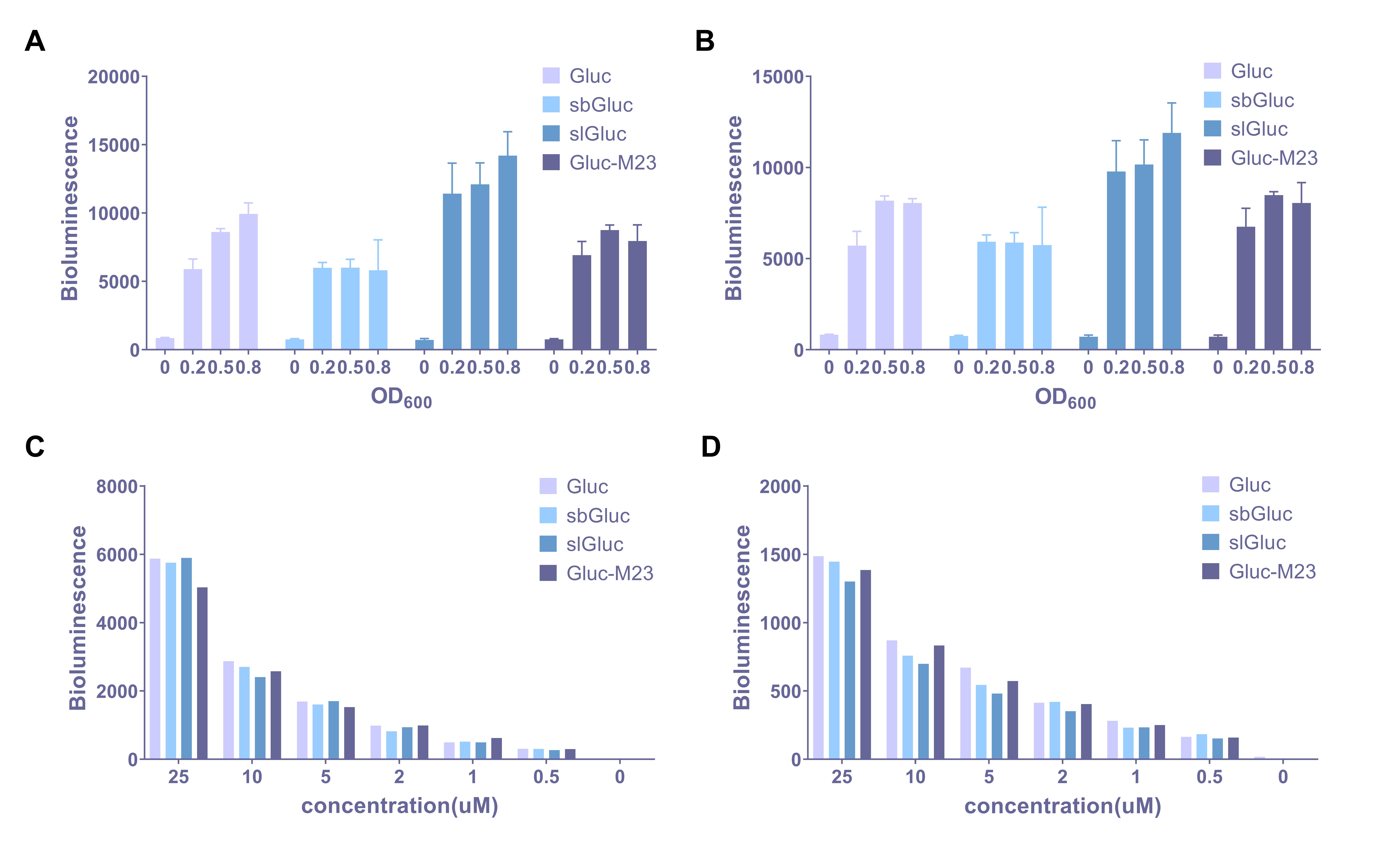


##### Figure S2. Optimization of combinatorial screening conditions for Gluc and mutant strains.

The OD_600_ of each bacterial culture was diluted to 0, 0.2, 0.5, and 0.8. After lysing the bacteria culture, the lysate was immediately mixed with the compound solution to test bioluminescence intensity, as shown in panels A and C. When maintaining the OD_600_ of all bacterial cultures at 0.8, different concentrations of CTZ and DeepBlueC were prepared and lysed. The lysate was then mixed with the compound solution to test bioluminescence intensity, as shown in panels B and D. (A) Bioluminescence intensity of each Gluc luciferase and mutant at different bacterial concentrations with CTZ as substrate. (B) Bioluminescence intensity of each Gluc luciferase and mutants at different bacterial concentrations with DeepBlueC as substrate. (C) Corresponding intensity of each Gluc luciferase to different concentrations of CTZ at the same bacterial concentration. (D) Corresponding intensity of each Gluc luciferase to different concentrations of DeepBlueC at the same bacterial concentration.


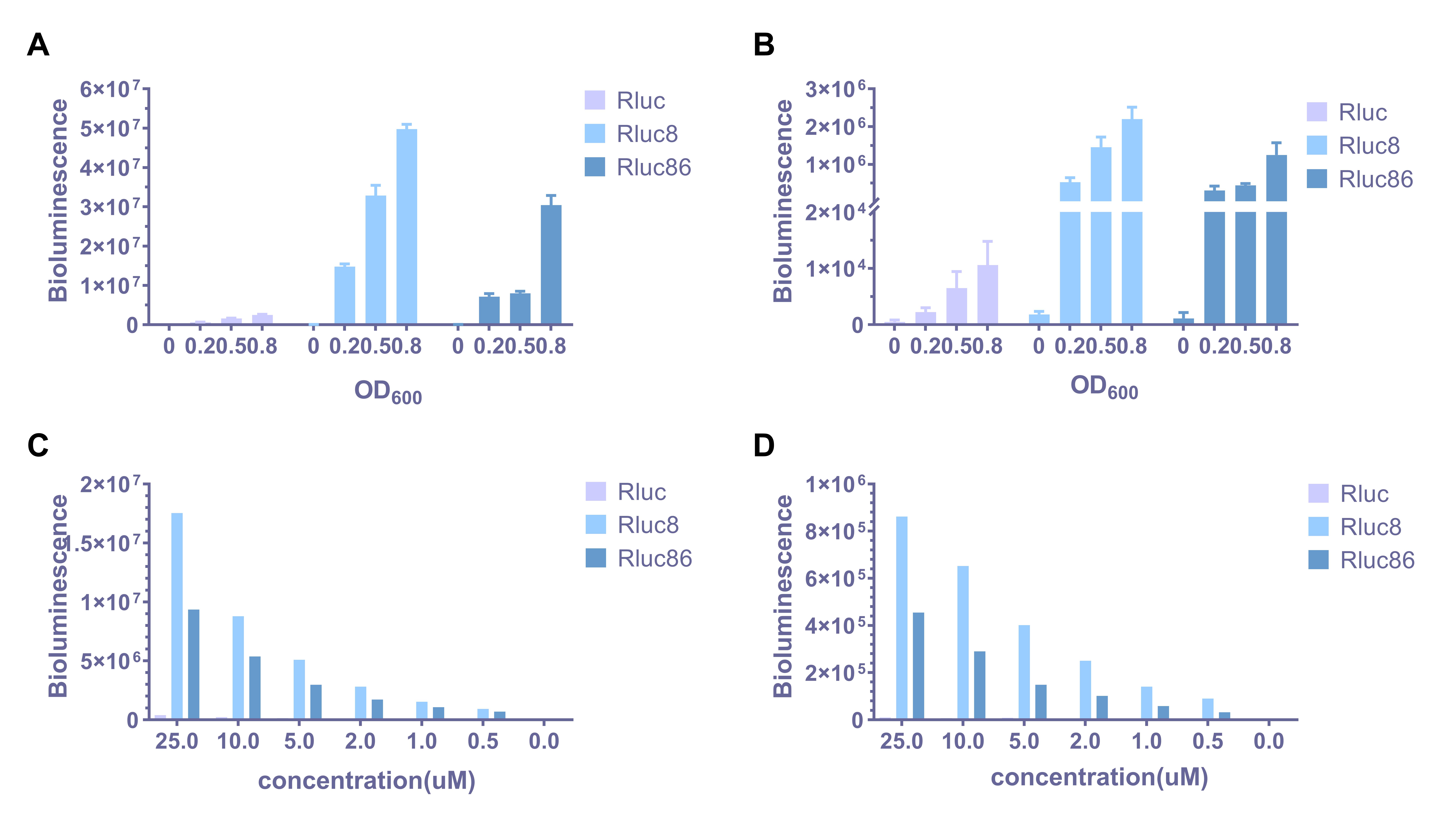


##### Figure S3. Optimization of combinatorial screening conditions for Rluc and mutant strains.

The OD_600_ of each bacterial culture was diluted to 0, 0.2, 0.5, and 0.8. After lysing the bacteria culture, the lysate was immediately mixed with the compound solution to test bioluminescence intensity, as shown in panels A and C. When maintaining the OD_600_ of all bacterial cultures at 0.8, different concentrations of CTZ and DeepBlueC were prepared and lysed. The lysate was then mixed with the compound solution to test bioluminescence intensity, as shown in panels B and D. (A) Bioluminescence intensity of each Rluc luciferase and mutant at different bacterial concentrations with CTZ as substrate. (B) Bioluminescence intensity of each Rluc luciferase and mutants at different bacterial concentrations with DeepBlueC as substrate. (C) Corresponding intensity of each Rluc luciferase to different concentrations of CTZ at the same bacterial concentration. (D) Corresponding intensity of each Rluc luciferase to different concentrations of DeepBlueC at the same bacterial concentration.


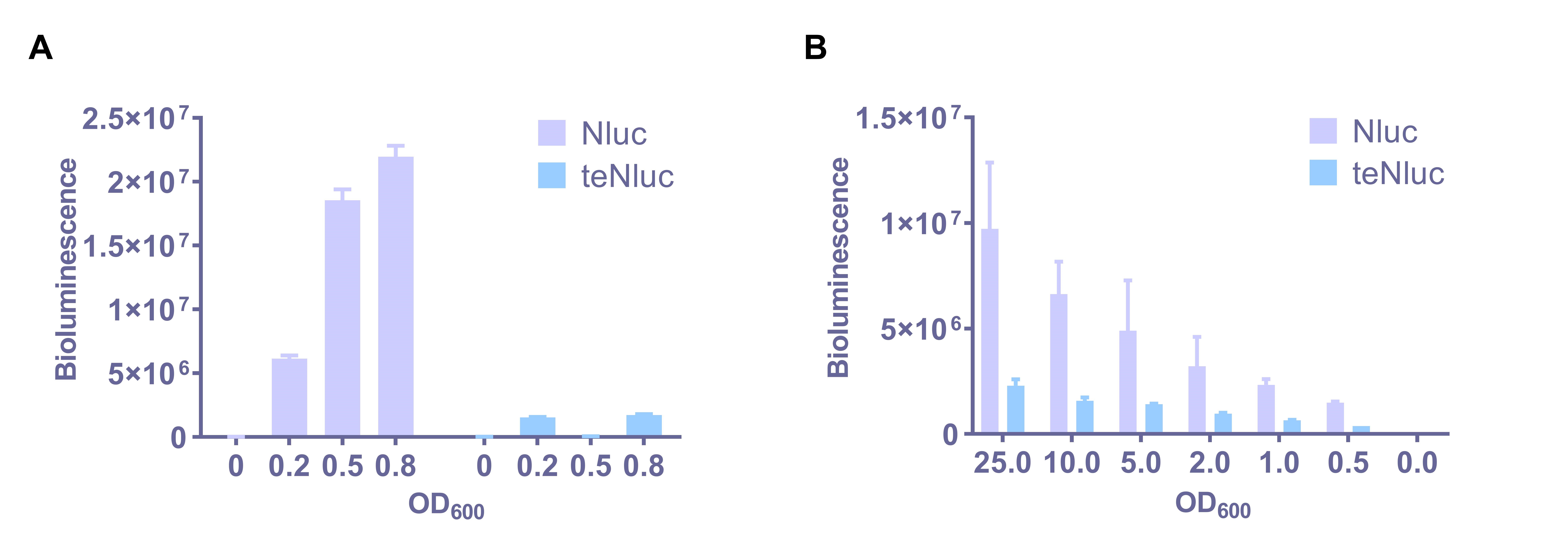


##### Figure S4. Optimization of screening conditions for Nluc and mutant strain combinations.

(A) Dilute the OD_600_ of the Nluc and teNluc bacterial cultures to 0, 0.2, 0.5, and 0.8, and immediately mix with the compound FRZ solution after lysis to test the bioluminescence intensity. (B) Maintain the bacterial cultures of each group diluted to OD_600_ of 0.8, prepared FRZ solutions with concentrations of 0.5, 1, 2, 5, 10, and 25 mM, then immediately mixed with the compound solution after lysis to test the bioluminescence intensity.


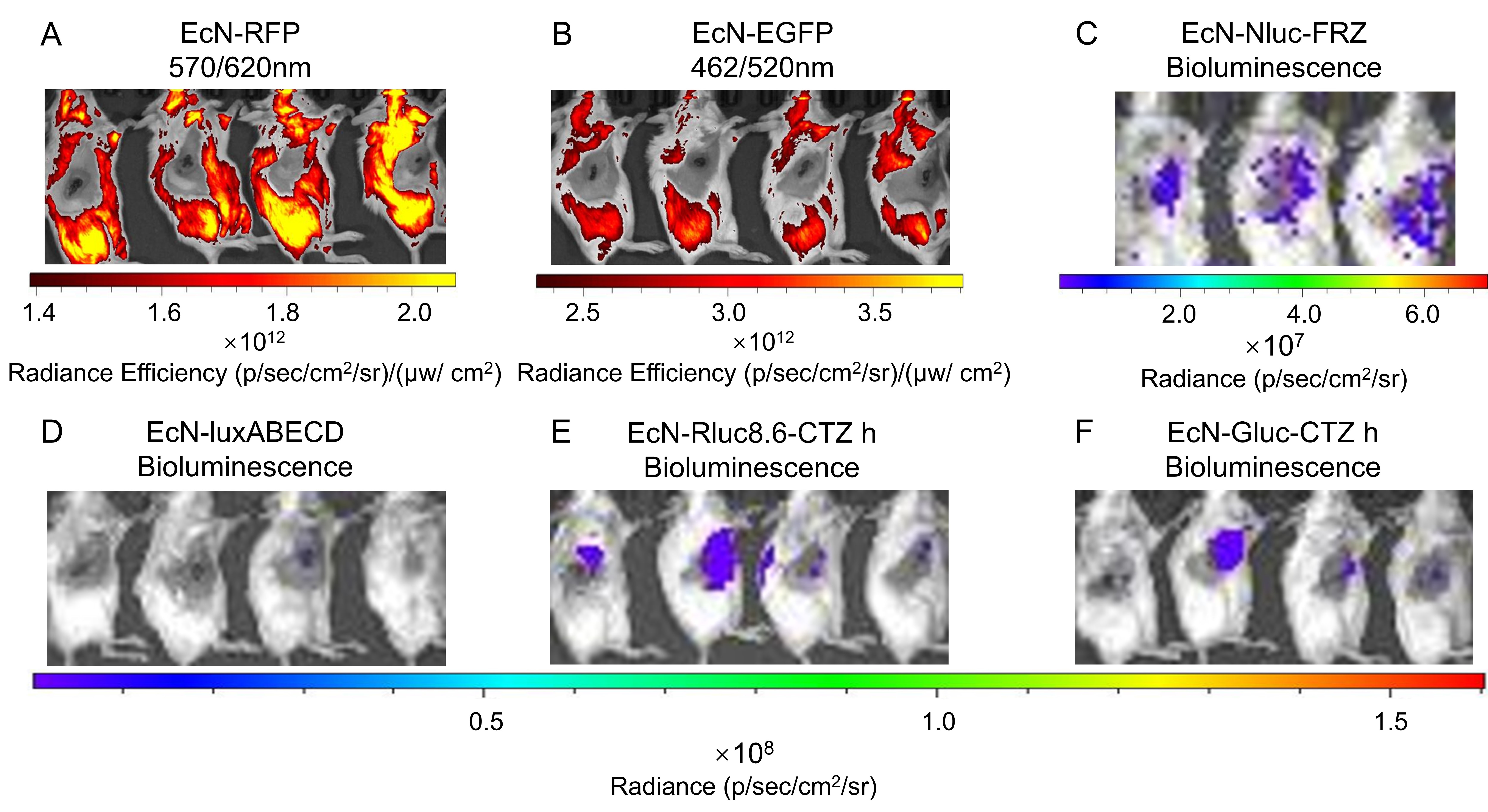


##### Figure S5. The contrast of fluorescent imaging and bioluminescence imaging

(A) The fluorescence imaging of EcN-RFP. (B) The fluorescence imaging of EcN-EGFP. (C) The bioluminescence imaging of EcN-Nluc-FRZ. (D) The bioluminescence imaging of EcN-luxABECD. (E) The bioluminescence imaging of EcN-Rluc8.6-CTZ h. (F) The bioluminescence imaging of Gluc-CTZ h.


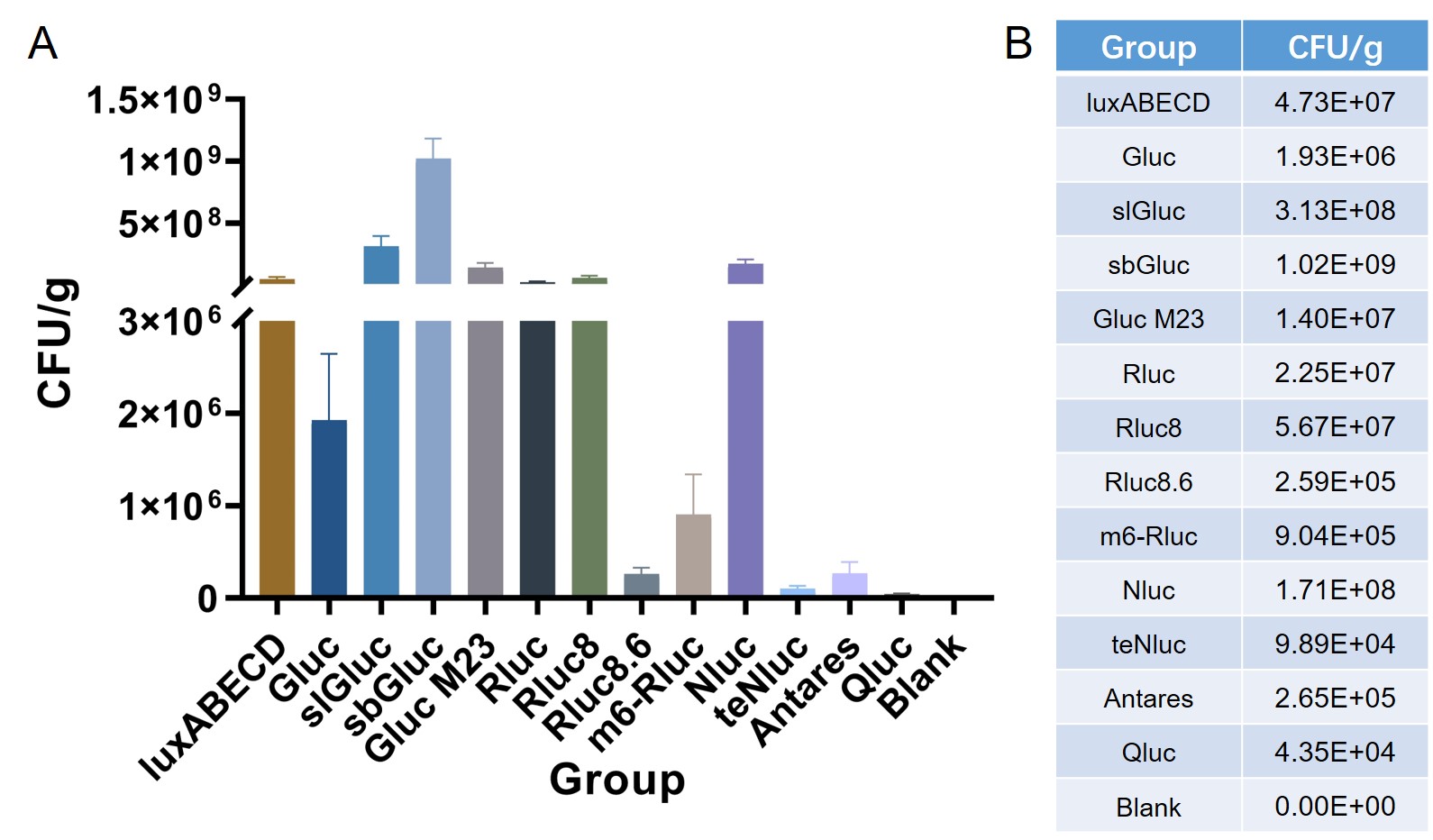


##### Figure S6. The CFU of engineered EcN colonized in the tumor.

(A) Forty-eight hours after the engineered EcN was injected via the tail vein, the tumor tissues were harvested, homogenized, and then diluted with PBS. Subsequently, the diluted samples were spread on LB agar plates containing chloramphenicol. After 16 hours, the colonies were counted to calculate the CFU. (B) The specific colonization status of each group was recorded.
